# The enzyme kinetics of branched‐chain fatty acid synthesis of metazoan fatty acid synthase

**DOI:** 10.1002/pro.70229

**Published:** 2025-07-16

**Authors:** Christian Gusenda, Kim Ochs, Ziheng Cui, Damian L. Ludig, Martin Grininger

**Affiliations:** ^1^ Institute of Organic Chemistry and Chemical Biology, Buchmann Institute of Molecular Life Sciences Goethe University Frankfurt Frankfurt am Main Germany

**Keywords:** biocatalysis, biosynthesis, branched‐chain fatty acids, enzyme kinetics, fatty acid synthase, ketoacyl synthase, methylmalonyl‐CoA, molecular dynamics

## Abstract

Branched‐chain fatty acids (BCFAs) exhibit enhanced oxidative stability, lower melting points, and reduced viscosity compared to straight‐chain fatty acids (StCFAs), making them valuable for biological systems and industrial applications. Previous studies have shown that metazoan fatty acid synthase (mFAS) can produce BCFAs through the incorporation of branched starter units and branched extender substrates such as methylmalonyl‐CoA (metmal‐CoA). However, the mechanistic and kinetic underpinnings of BCFA biosynthesis in metazoans remain poorly understood. Here, we address this by characterizing the kinetic parameters of mFAS‐catalyzed BCFA synthesis using NADPH consumption assays and analyzing the synthesized products via GC–MS. These experiments revealed a lower turnover number of mFAS with metmal‐CoA and a shift toward medium‐chain fatty acids compared to the native StCFA biosynthesis with malonyl‐CoA. The ketoacyl synthase (KS) kinetic measurements revealed low elongation rates, indicating that the KS domain dictates the substrate specificity and speed of BCFA production. To gain a better understanding of the reaction mechanism, we performed molecular dynamic simulations, which revealed key KS: substrate interactions by conserved threonine residues. These insights support a refined decarboxylative condensation mechanism and illuminate how substrate specificity arises within mFAS. These findings offer a foundation for future protein engineering and inhibitor design strategies.

## 1 INTRODUCTION

The most commonly found fatty acids (FAs) in all organisms are straight‐chain fatty acids (StCFAs), which serve various functions, including roles in membranes, energy storage, and signaling pathways (Calder, 2015; van Meer et al., 2008). In addition, branched‐chain fatty acids (BCFAs), although mostly present in low concentrations, also serve important biological functions—for example, by regulating membrane fluidity (Budin et al., 2018). In humans, BCFAs have been identified in milk (Insull & Ahrens, 1959), blood (James et al., 1959), sebum (James & Wheatley, 1956), meibum (Andrews, 1970; Harvey et al., 1987), vernix (Ran‐Ressler et al., 2008), and adipose tissue (Horning et al., 1961). Their function in humans is largely unknown, yet they have been associated with gut health (Ran‐Ressler et al., 2008; Ran‐Ressler et al., 2011), cancer control (Wongtangtintharn et al., 2004; Yang et al., 2000), obesity, and hypoxia (Wallace et al., 2018), among other processes (Gozdzik et al., 2023). Moreover, BCFAs exhibit higher oxidative stability, lower melting points, and lower viscosity than their straight‐chain counterparts, which make them a promising target for biolubricants and biofuels (Bai et al., 2019; Wagner et al., 2001). They also show great potential as food additives and aroma compounds, both of which make the biotechnological production of BCFAs desirable (Fan et al., 2023; Zhou et al., 2025). Despite their known physiological and industrial importance, the mechanisms underlying BCFA synthesis in metazoans are not thoroughly understood.

**FIGURE 1:**
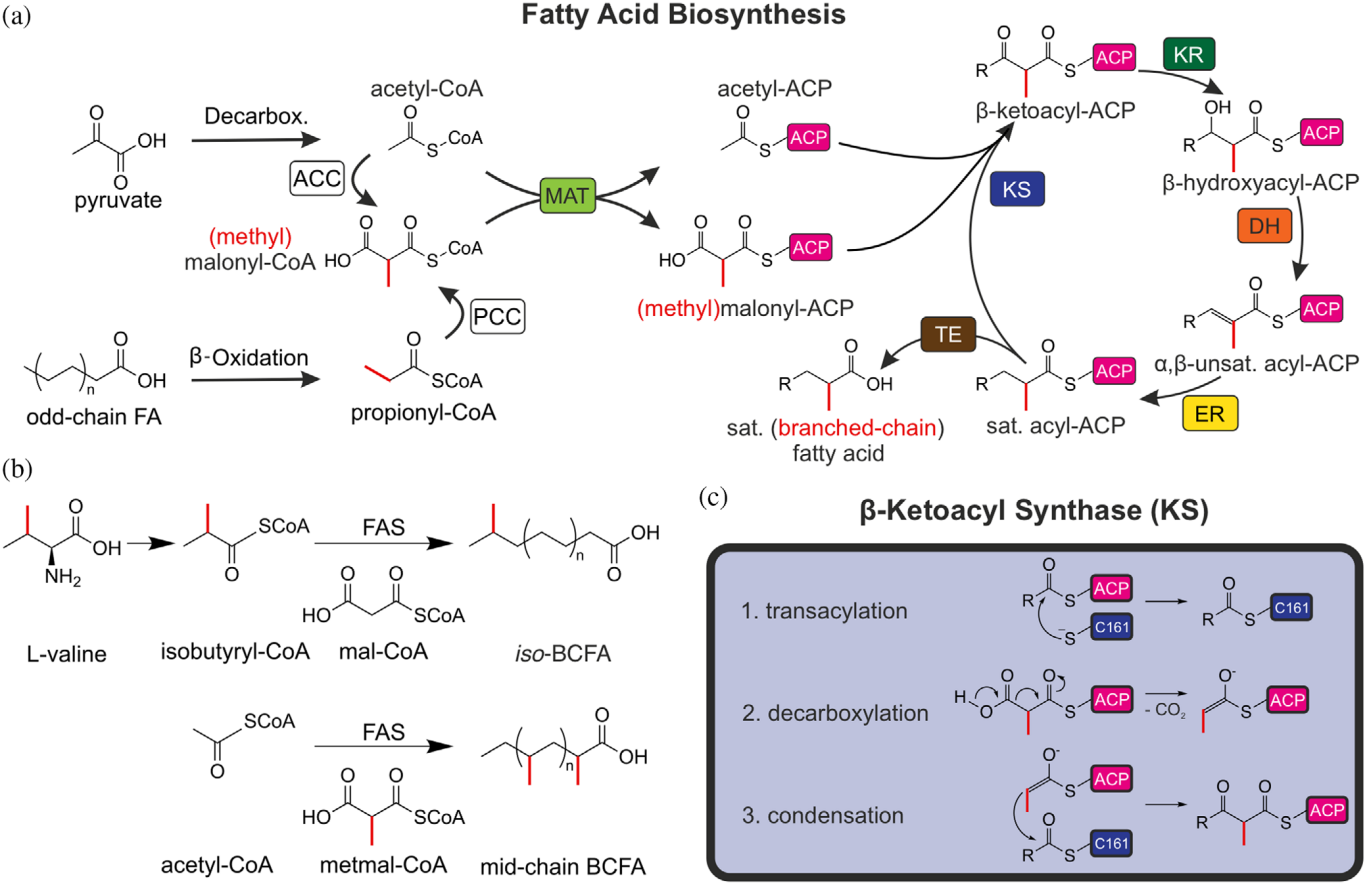
BCFA metabolism. (a) Fatty acid (FA) biosynthesis. Generation of substrates for fatty acid synthase (FAS) starts from central metabolism, like glycolysis that leads through pyruvate to acetyl‐CoA or β‐oxidation that leads to propionyl‐CoA. The extender (methyl)malonyl‐CoA is synthesized by acetyl‐CoA carboxylase (ACC) or propionyl‐CoA carboxylase (PCC), respectively. During the FA cycle, starter and extender substrates are sequentially transferred to the acyl carrier protein (ACP), which shuttles the substrates and intermediates between the catalytic domains. The elongation reaction is catalyzed by the ketoacyl synthase (KS) domain, producing β‐ketoacyl‐compounds. During the processing steps, the intermediate is reduced by the β‐ketoacyl reductase (KR) to β‐hydroxyacyl‐ACP, dehydrated by the dehydratase (DH) to α,β‐unsaturated acyl‐ACP, and further reduced by the enoyl reductase (ER) to saturated acyl‐ACP. The cycle is repeated until a certain chain length (canonically C16, palmitic acid) is reached, and the product is released by the thioesterase (TE). (b) Origin of branching. Pathway to *iso*‐ and *anteiso*‐branched‐chain fatty acids (BCFAs) based on branched‐chain amino acids metabolism and the extension with malonyl units by FAS shown on the example of L‐valine elongation to *iso*‐BCFA. The pathway to mid‐chain mono‐ and multi‐BCFAs is based on the extension with methylmalonyl units. (c) The elongation reaction proceeds in a double displacement reaction, which is comprised of two sub‐reactions. A transacylation reaction transfers a starter unit to the active site cysteine (C161 in murine FAS), called ping reaction (C1). A subsequent decarboxylative Claisen condensation covalently couples the previously bound starter unit to an extender substrate, called Pong reaction (C2 and C3).

Recent studies show that BCFAs can not only be consumed with our diet but also de novo synthesized in metazoans by the same multidomain fatty acid synthase (FAS) that catalyzes the synthesis of StCFAs, albeit at substantially lower rates (Figure 1a) (Buckner et al., 1978; Dewulf et al., 2019; Ran‐Ressler et al., 2014; Seyama et al., 1981; Wallace et al., 2018).

In general, these BCFAs can be classified into mono‐BCFAs and multi‐BCFAs, among which the most abundant are monomethyl‐branched *iso*‐ and *anteiso*‐FAs, exhibiting a branching at the penultimate and antepenultimate carbon atom (Figure 1b) (Horning et al., 1961; Kaneda, 1991). Multimethyl‐ and mid‐chain branched FAs have also been found in metazoans, for example, in the preen gland of birds (Jacob & Poltz, 1974), human vernix (Ran‐Ressler et al., 2008), sebum (Nicolaides, 1974), and adipocytes (Dewulf et al., 2019). The biosynthetic pathway to *iso*‐ and *anteiso*‐FAs on one hand and the synthesis of mid‐chain branched and multimethyl branched FAs on the other differ in the origin of the branch. While branching at the penultimate and antepenultimate carbon atom requires a branched starter unit, substituting acetyl‐CoA, branching in the middle of the chain requires the incorporation of a branched malonyl‐CoA (mal‐CoA) derivative (Figure 1b). It has been shown that the starter units isovaleryl‐CoA, isobutyryl‐CoA, and 2‐methylbutyryl‐CoA, deriving from the degradation of the branched‐chain amino acids (BCAAs) leucine, valine, and isoleucine, respectively, are directly utilized during the synthesis of *iso*‐ and *anteiso*‐BCFA (Wallace et al., 2018). The mid‐chain branching, on the other hand, can be introduced by methylmalonyl‐CoA (metmal‐CoA) and ethylmalonyl‐CoA (Dewulf et al., 2019). Yet, the molecular details of BCFA biosynthesis by mFAS remain unclear.

So far, one of the major factors influencing BCFA synthesis seems to be the supply of metmal‐CoA. The cellular levels of metmal‐CoA are subject to different regulatory mechanisms. In metazoans, (*S*)‐metmal‐CoA is synthesized during the catabolism of certain amino acids and odd‐chain FAs, which yields propionyl‐CoA at first (Figure 1a). This intermediate is carboxylated by the propionyl‐CoA carboxylase (PCC), resulting in (*S*)‐metmal‐CoA, which is further converted by multi‐step catalysis to eventually enter the tricarboxylic acid cycle as succinyl‐CoA. A decreased metmal‐CoA catabolism is associated with pathological conditions (Bikker et al., 2006; Narasimhan et al., 1996). Hence, the question arises if metazoan cells are indeed capable of accumulating enough extender substrates for BCFA synthesis.

In actinomycetes, several pathways have been identified that can lead to the accumulation of (*S*)‐metmal‐CoA (Chan et al., 2008), of which the carboxylation of propionyl‐CoA is believed to be its predominant origin (Bramwell et al., 1996). These organisms are well‐known producers of various methylated polyketides that derive from the catalysis of polyketide synthases (PKSs), a close relative of the FAS. In contrast to bacteria accumulating metmal‐CoA for polyketide biosynthesis, in metazoans no methylated polyketides were identified, thus diminishing any apparent need for metmal‐CoA for the anabolic metabolism. However, recent research on mollusks revealed an animal FAS‐like polyketide synthase (AFPKS), which catalyzes the biosynthesis of multi‐methylated polyketides derived from metmal‐CoA (Torres et al., 2020). Although it seems unlikely that metazoan cells accumulate substantial concentrations of metmal‐CoA at all times, specialized cells might be able to utilize this compound for polyketide or BCFA biosynthesis.

Here, we provide in vitro data for BCFA synthesis by examining the murine FAS as a model enzyme. Specifically, we report mFAS kinetics and product profiles for multimethyl‐ and mid‐chain branched FAs. In our previous study, we were able to determine the kinetic characteristics of the ketoacyl synthase (KS)‐catalyzed elongation reaction of saturated FAs with mal‐acyl carrier protein (ACP) that leads to StCFAs (Gusenda et al., 2025). This study has indicated that the KS domain is the determining domain in substrate discrimination during the natural elongation reaction, which likely arises from unproductive acyl‐ACP binding during the transacylation step (Figure 1c). The question arises whether the KS might have an influence on extender substrate discrimination as well. We therefore determined the reaction kinetics of the elongation with metmal‐ACP and performed molecular dynamic (MD) simulations to gain a molecular understanding of KS: substrate interactions.

## 2 RESULTS

### 2.1 BCFA production by mFAS

Naturally, the key role of the mFAS is the production of StCFAs, which is performed at a rate of about three condensations per second (Carlisle‐Moore et al., 2005; Rittner et al., 2019). However, the specific activity of the mFAS toward the production of BCFAs is two to three orders of magnitude lower compared to the production rates of StCFAs (Buckner et al., 1978). The kinetic constants *k*_cat_ and *K*_m_ of BCFA synthesis have not been determined yet.

**FIGURE 2:**
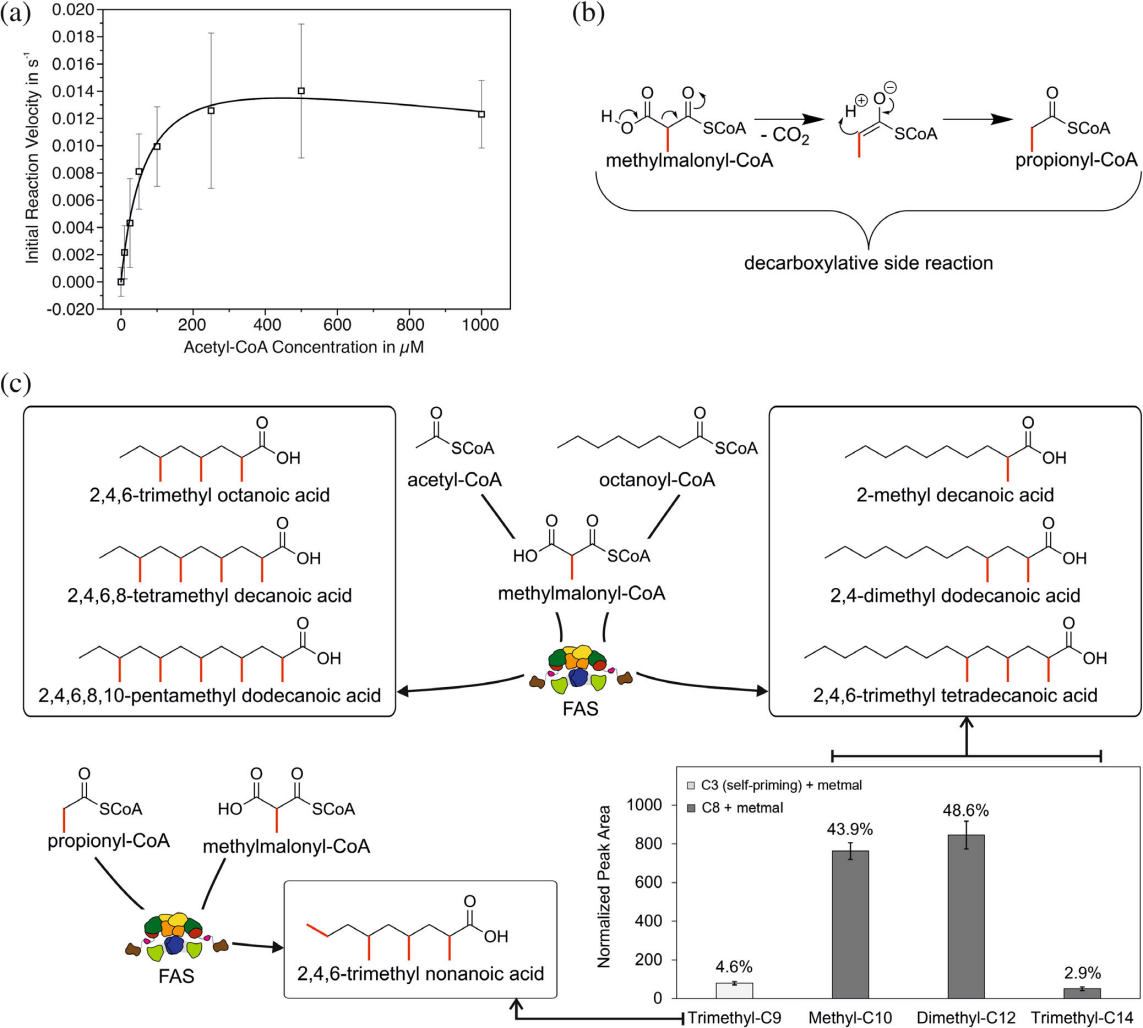
Metazoan fatty acid synthase (mFAS) kinetics and product output with methylmalonyl‐CoA (metmal‐CoA) as elongation substrate. (a) Initial reaction velocity of mFAS (measured by NADPH fluorescence) during acetyl‐CoA titration. Means were fitted under the assumption of substrate inhibition (Equation S3). Data points reflect means of three biological replicates. Error bars reflect propagated standard deviation. (b) Decarboxylative side reaction from metmal‐CoA to propionyl‐CoA. (c) Products of mFAS with (*R,S*)‐metmal‐CoA and acetyl‐CoA or octanoyl‐CoA. Molecular structures as analyzed by GC–MS and their proposed formation pathway. Acetyl‐derived products are shown in the left box, octanoyl‐derived products are shown in the right box, and self‐priming‐derived product is shown in the lower box. Bar plot represents product distribution of mFAS with octanoyl‐CoA and (*R*,*S*)‐metmal‐CoA. The bars represent peak areas normalized to the peak area of the internal standard, whereas numbers above the bars indicate ratios of the individual products to total products. The products were enzyme‐catalytically synthesized overnight and transformed into fatty acid methyl esters before monitoring in GC–MS. The bars represent means of three biological replicates, with error bars representing the propagated standard deviation.

For the determination of the kinetic constants of the multidomain enzyme, we measured the nicotinamide adenine dinucleotide phosphate (NADPH) consumption of the integrated β‐ketoacyl reductase and enoyl reductase domains as described previously (Rittner et al., 2019). During the course of this assay, the native fatty acid synthesis is performed as described in the previous section (Figure 1a). The titration of acetyl‐CoA in the presence of (*RS*)‐metmal‐CoA shows decreasing turnover at high concentrations, which indicates substrate inhibition (Figures 2a and S1). In agreement, the *F*‐test confirmed that the substrate inhibition equation (Equation S3) fits the measured activity significantly better than the Michaelis–Menten equation (Equation S1). This phenomenon is expected as the substrates likely compete in the malonyl‐acetyl transferase (MAT) binding pocket, as was seen previously for mal‐CoA (Rittner et al., 2019). Interestingly, we observed increasing activity with increasing metmal‐CoA in the absence of starter substrates. This likely derives from decarboxylation of metmal‐CoA (or metmal‐ACP) to propionyl‐CoA (or propionyl‐ACP) and subsequent “self‐priming” (Figures 2b and S2) leading to odd‐chain FAs, as suggested previously (Buckner et al., 1978). Due to the significant overlap of the self‐priming side reaction with the main reaction at high extender concentrations, accurate kinetic characterization of the primary pathway becomes unfeasible. Instead, apparent constants were determined at 100 μM (*RS*)‐metmal‐CoA and varying acetyl‐CoA concentrations (Table 1, Figure 2a).

**TABLE 1:** Kinetic values of metazoan fatty acid synthase (mFAS) and ketoacyl synthase (KS). Apparent kinetic constants determined by the NADPH consumption assay (mFAS activity) for acetyl‐CoA and methylmalonyl‐CoA (metmal‐CoA). Apparent kinetic constants determined by the MabA assay (KS activity) for decanoyl‐acyl carrier protein (ACP) and metmal‐ACP. The values are given as mean ± standard error of the parameter estimate.

Enzymatic property	Fatty acid cycle (total mFAS)	Chain elongation (KS single domain)
Turnover number *k*_cat_	0.0176 ± 0.0014 s^−1^/1.1 ± 0.1 min^−1^	0.0116 ± 0.0006 s^−1^/0.70 ± 0.04 min^−1^
Half‐saturation constant *K*_m_/*K*′	69 ± 12 μM	23.7 ± 1.4 μM
Hill coefficient *h*	‐	3.3 ± 0.5
Inhibition constant *K*_*i*_	2.9 ± 1.2 mM	270 ± 20 μM

The *k*_cat_ of the BCFA biosynthesis is around 170 times lower compared to StCFA synthesis (Carlisle‐Moore et al., 2005; Rittner et al., 2019). This turnover rate is comparable with that of evolutionarily related PKSs (Aldrich et al., 2005; Buyachuihan et al., 2023; Moriguchi et al., 2010; Robbins et al., 2016), which indicates that mFAS is able to produce BCFAs with the same rate as related enzymes produce secondary metabolites. The *K*_m_ of the mFAS toward acetyl‐CoA in the reaction with metmal‐CoA is around 350 times higher compared with mal‐CoA, leading to *k*_cat_/*K*_m_ with four orders of magnitude difference.

The products of the mFAS in the presence of acetyl‐CoA and metmal‐CoA (without mal‐CoA supply) were analyzed using gas chromatography‐mass spectrometry (GC–MS) (Figure S3). The main product of multi‐BCFA synthesis is 2,4,6,8‐tetramethyl decanoic acid (Figure 2c), which is consistent with previous reports (Buckner et al., 1978). 2,4,6‐Trimethyl octanoic acid and 2,4,6,8,10‐pentamethyl dodecanoic acid are found in smaller amounts. Odd‐chain branched FAs that are synthesized from self‐priming with propionyl units could not be unequivocally determined in our measurement but were identified in small quantities previously (Buckner et al., 1978).

As indicated by previous experiments (Dewulf et al., 2019), and the increased elongation rates of medium‐chain FAs with metmal‐CoA (see Section 2.2), the synthesis of BCFAs in cells likely occurs through a mixed use of mal‐ and metmal‐CoA. Hence, we analyzed the products of the mFAS in the presence of octanoyl‐CoA (mimicking preceded malonyl elongations) and metmal‐CoA, which revealed 2‐methyl decanoic acid and 2,4‐dimethyl dodecanoic acid as main products (Figure 2c). These findings suggest that, rather than proceeding through the full seven elongation cycles required to produce the canonical palmitic acid, mFAS carries out a reduced number of elongations with metmal‐CoA as an extender substrate. Specifically, acetyl‐CoA is elongated up to five times (mostly four), while octanoyl‐CoA undergoes up to three elongations (mostly one and two), corresponding to four or five total elongation cycles when traced back to acetyl‐CoA as the starter unit. As the chain length control in multidomain FASs is the result of the interplay of the elongating KS catalysis and the terminating thioesterase catalysis (Gajewski, Buelens, et al., 2017; Gajewski, Pavlovic, et al., 2017; Heil et al., 2019), the reason for the reduced chain lengths might lie in the low rate of acyl chain elongation. This means that the elongation reaction is so slow that the probability of “premature” termination is increased. This explanation is corroborated by the analysis of the KS kinetics in the next section. Given that longer products are received with octanoyl‐CoA as the priming substrate, multimethyl branches seem to be less well accommodated in the KS binding pocket than non‐ or mono‐branched intermediates.

Additionally, we could identify the odd‐chain fatty acid 2,4,6‐trimethyl nonanoic acid, which derives from the priming with propionyl moieties (Figure 2b,c). This side reaction contributes around 5% to the overall products during the synthesis of multi‐BCFAs with octanoyl‐CoA (Figure 2c). Comparing BCFAs from acetyl‐ and octanoyl‐CoA, it appears that self‐priming products are more frequently found when octanoyl‐CoA is supplied, although the process should be independent of the starter substrate. We speculate that self‐priming rates might therefore trace back to the readiness of the unoccupied KS to perform decarboxylation. As the final product from octanoyl‐CoA is produced within only two elongation cycles, the system is unloaded more frequently during the synthesis starting from octanoyl‐CoA than during the synthesis from acetyl‐CoA. Decarboxylation and priming with propionyl moieties can occur in these unoccupied KS binding pockets of unloaded systems. The contribution of this side reaction is not evident from product spectra in the canonical even‐numbered StCFA (C16‐ and C18‐FAs) synthesis, as the product of malonyl decarboxylation is the naturally used acetyl starter.

### 2.2 KS‐catalyzed elongation kinetics

Given that the turnover number for BCFA production is about 150 times lower than the production of StCFAs, the question arises, which domain is responsible for the discrimination of the methymalonyl extender unit. It was shown that the MAT domain is in principle able to load this alternative extender onto the ACP with similar activities as the commonly used malonyl moiety, proving that the MAT does not mediate substrate specificity (Rittner et al., 2018). In our previous study, we were able to determine the kinetic characteristics of the KS‐catalyzed elongation reaction of saturated FAs with mal‐ACP that leads to StCFAs using the enzyme‐coupled β‐ketoacyl reductase from *Mycobacterium tuberculosis (*MabA) assay (Gusenda et al., 2025). The results indicated that the KS domain determines the fidelity of fatty acid biosynthesis by imposing substrate selectivity in the step of transferring the acyl‐moiety from ACP to the KS domain (transacylation step, Figure 1c). Given the decreased rates in fatty acid biosynthesis with metmal‐CoA, the question arises whether the KS might have an influence on extender substrate discrimination as well.

**FIGURE 3:**
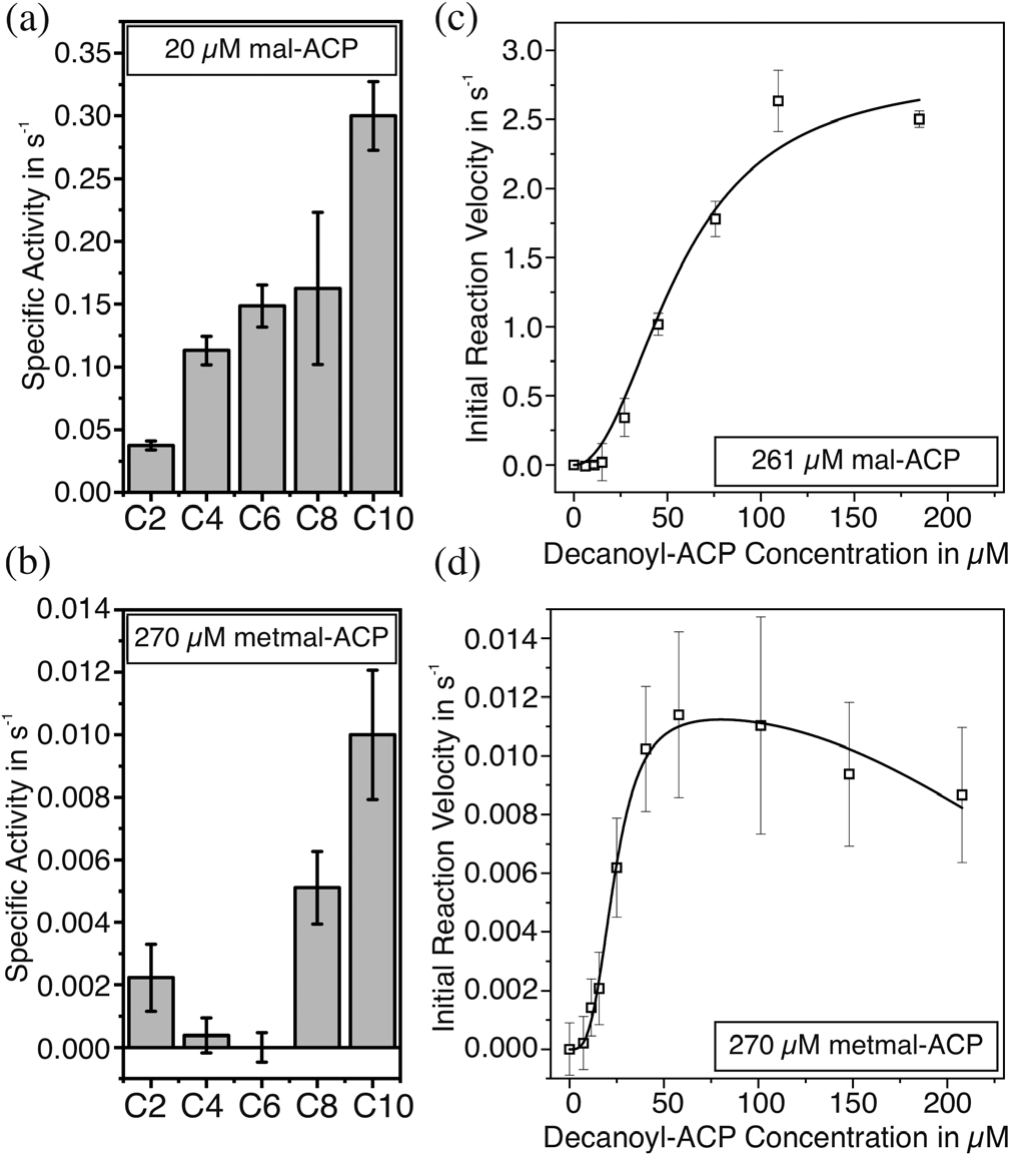
Elongation activities of the ketoacyl synthase (KS) determined by the enzyme‐coupled MabA assay. (a) The specific activity of the KS domain was recorded for acyl‐acyl carrier protein (ACPs) of saturated fatty acids with chain lengths of two to 10 carbon atoms and mal‐ACP as extender substrate. (b) Specific activity of the KS domain for different acyl‐ACPs elongated with metmal‐ACP. (c) Titration curve of the initial reaction velocity at different decanoyl‐ACP concentrations at a constant mal‐ACP concentration. These data were published previously (Gusenda et al., 2025). (d) Titration curve of the initial reaction velocity at different decanoyl‐ACP concentrations at a constant metmal‐ACP concentration. Data of (c) and (d) were fitted with Equation (S4) using OriginLab. Bars and data points represent the means of three biological replicates, and errors represent the propagated error of the standard deviation.

Previously, in employing starter substrates of different chain lengths (C2–C10) in the KS elongation assay, we demonstrated that KS activity increases with chain length when mal‐ACP serves as the extender substrate (Figure 3a). Here, utilizing metmal‐ACP as the extender substrate, we found that this pattern is not replicated. The activity decreases from two to six carbon atoms and shows a steep increase toward substrates with a chain length of eight and 10 carbon atoms (Figure 3b). Of note, as the combination of both starter and extender substrate influences the elongation pattern, these data indicate that the identity of the starter substrate influences the binding pocket of the extender substrate. Specifically, the chain length profile suggests that there is a higher chance of the mFAS to incorporate methyl branching at the sixth carbon atom of palmitic acid (elongation of C10‐ACP) than on the 10th carbon atom (elongation of C6‐ACP). Due to the absence of in vivo data on cellular BCFA synthesis, it remains unclear to what extent our enzymatic findings reflect physiological conditions. While the BCFA profile of adipocytes was determined previously, the detailed ratio of branch positions could not be determined for technical reasons (Dewulf et al., 2019).

By comparing specific activity for the elongation of C8 and C10 with mal‐ and metmal‐ACP, respectively, the incorporation of branching (BCFA synthesis) is approximately 30 times slower than the canonical elongation to the non‐branched StCFAs.

To receive extended and quantitative information on the elongation with metmal‐CoA versus mal‐CoA, we aimed at comparing Michaelis–Menten kinetics for elongation of decanoyl‐ACP with both extender substrates (Figure 3c‐d). Data on the elongation of decanoyl‐ACP with mal‐ACP were previously reported by us (Figure S4) (Gusenda et al., 2025). In the titration of decanoyl‐ACP with metmal‐ACP, we observed a sigmoidal rise of the reaction velocity, which indicates enzyme cooperativity as previously seen (Gusenda et al., 2025; Rittner et al., 2020; Schultz et al., 2025). At higher substrate concentrations the reaction velocity drops, which was also observed previously for mFAS (Rittner et al., 2019). This pattern is not sufficiently well explained by either an equation that takes cooperativity into account (Equation S2) or by an equation that takes solely substrate inhibition into account (Equation S3), but is well represented by a combined model (Figures 3d and S5, Equation S4).

The apparent *k*_cat_ of the titration with metmal‐ACP is 276 times lower compared with the *k*_cat_ of the C10‐ACP/mal‐ACP reaction, which again manifests the high preference of the KS for mal‐ACP. The comparison of *k*_cat_ for the FA cycle with acetyl‐CoA as starter, representing the overall activity of mFAS, and the single elongation with C10‐ACP, which reflects the optimal activity of the KS domain alone (1.1 vs. 0.7 min^−1^, Table 1), suggests that indeed the KS specificity is limiting BCFA synthesis. As the elongation assay includes saturated starter substrates, which are known to be transacylated with high rates (first reaction step, Figure 1c) (Insull & Ahrens, 1959), the activity impairment of the KS originates from the decarboxylative condensation (second/third reaction step, Figure 1c).

The half‐saturation constant *K*′_C10_ is about two times higher for the reaction with mal‐ACP. The resulting enzymatic efficiency ratio of the reaction with mal‐ACP and metmal‐ACP is calculated as $k_{\mathrm{cat}}/K_{C10}^{\prime }\left(\mathrm{mal}\right)/k_{\mathrm{cat}}/K_{C10}^{\prime }\left(\text{metmal}\right)=108$. The discrimination against metmal‐ACP is therefore higher than the previously determined discrimination against crotonyl‐ACP, an intermediate of the FA cycle (Gusenda et al., 2025). According to the Transition State Theory (Laidler & King, 1983), the transition state energy difference that leads to a difference in specificity is ΔΔ*G* = 14 kJ mol^−1^, which corresponds to a moderate non‐covalent interaction. The apparent Hill coefficient h is consistent with previously reported values (Gusenda et al., 2025). The inhibition constant *K*_*i*_ = 270 μM indicates moderate competition of the starter and extender substrate in the binding pocket of the KS.

As the MAT does not efficiently select the extender substrate and hence cannot account for the product fidelity of mFAS (Rittner et al., 2018), the KS is presented almost equally with mal‐ACP or metmal‐ACP. This means BCFA synthesis in vivo is directly related to KS preference of mal‐ACP over metmal‐ACP and highly depends on the availability of extender substrates. Indeed, it was shown that the increase of metmal‐CoA and decrease of mal‐CoA promote the synthesis of BCFAs (Buckner et al., 1978; Buckner & Kolattukudy, 1975; Dewulf et al., 2019; Kim & Kolattukudy, 1978). Thus, the KS dictates the product fidelity regarding correct extender substrate selection, as previously shown for starter and intermediate substrates (Gusenda et al., 2024). The same rule might apply to polyketide synthesis by AFPKSs, which show metmal‐CoA specificity that is not mediated by their AT domain (Schubert et al., 2025). This is a remarkable difference to PKS, for which it was shown that the AT domain strictly selects for the correct extender substrate (Khosla et al., 1999).

### 2.3 Molecular dynamic simulations

To gain a better understanding of the molecular details of the methylmalonyl discrimination, we performed MD simulations with the octanoyl‐bound KS domain (PDB: 6ROP) (Rittner et al., 2020) and three possible extender substrates: mal‐phosphopantetheine (Ppant), (*S*)‐metmal‐Ppant, and (*R*)‐metmal‐Ppant. As the decarboxylation is the irreversible step in substrate elongation, we suggest that extender substrate binding and decarboxylation, but not the carbon–carbon‐bond‐forming condensation (Figure 1c) dictate extender substrate specificity. Thus, MD simulations on the substrate:KS binding before the decarboxylation can give insight into the specificity of the KS for the extender substrate.

**FIGURE 4:**
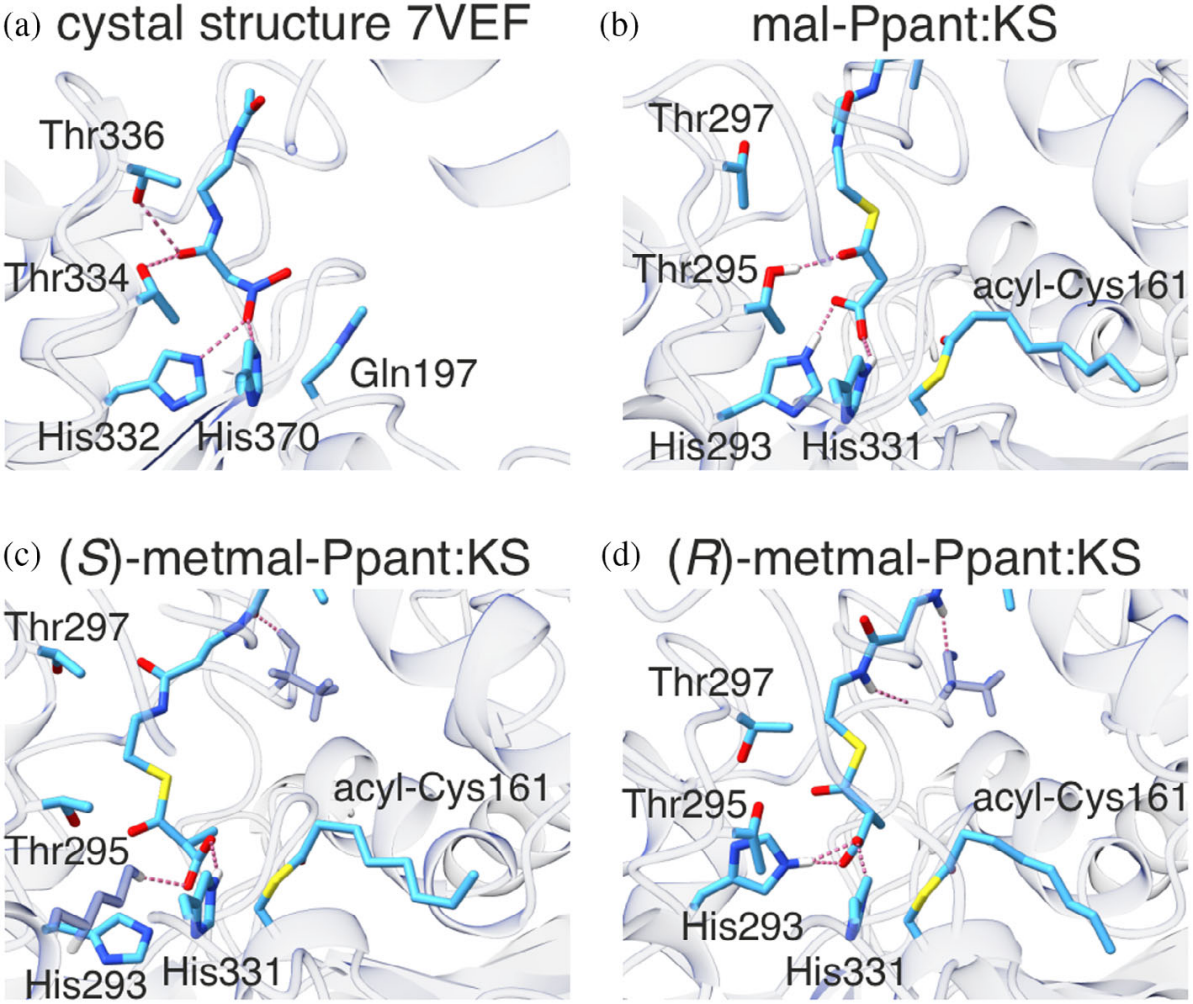
Center structures from *K*‐means clustering of all molecular dynamic trajectories. (a) Crystal structure of ketoacyl synthase (KS)‐like carboxylase GfsA KS^Q^ (PDB: 7VEF) (Chisuga et al., 2022). (b–d) Representative conformation (center structures) of mal‐Ppant:octanoyl‐KS, (*S*)‐metmal‐Ppant:octanoyl‐KS and (*R*)‐metmal‐Ppant:octanoyl‐KS. Catalytically relevant residues are labeled and shown in sticks. H‐bonds are shown in pink dashed lines. The image was generated with ChimeraX (Meng et al., 2023).

We used SwissDock to generate an initial substrate conformation for further MD refinement (Figure S6) (Bugnon et al., 2024; Eberhardt et al., 2021). Interestingly, we did not receive any docked structure that reflects previous proposals, which suggest that the thioester oxygen is coordinated by either the equivalent to His331 or both conserved histidine residues (Heath & Rock, 2002; Lee & Engels, 2013; Paiva et al., 2021). The generated docking structures were evaluated by comparing them to the crystal structure of the substrate analog nitroacetyl pantetheinamide in the KS‐like decarboxylase GfsA KS^Q^ domain from *Streptomyces graminofaciens* (PDB: 7VEF, Figure 4a) (Chisuga et al., 2022). The following MD simulations were carried out in five replicas of 100 ns, which showed a stable protein backbone (Figure S7). The substrate, on the other hand, showed large conformational variability, which led us to sort all conformations of the five trajectories by *K*‐means clustering (Brandman et al., 2011; Pedregosa et al., 2011; Stamatelou, 2020) to determine the most probable conformational states (Figure S8).

The center structure of each cluster was evaluated by consulting essential features that are integral to the current mechanistic understanding of the decarboxylative condensation reaction (Figure 4a), which integrates recent insight received on the KS^Q^‐mediated decarboxylation reaction: (Chisuga et al., 2022; Paiva et al., 2021) (i) The hydrogen bonds of conserved threonines (Thr295 and Thr297) and the thioester oxygen, as well as hydrogen bonds of histidines (His293 and His331) and the carboxyl group of metmal‐ACP are established, respectively. (ii) The optimal angle in the transition state of decarboxylation is about 90° between the thioester C‐O bond and the cleavable Cα‐Cβ bond (Bach & Canepa, 1996). (iii) The distance between the C2 atom of the (methyl)malonyl‐substrate and the C1 atom of the acyl substrate is relevant for the nucleophilic attack/condensation reaction.

Both the dihedral angle (ii) and substrate distance (iii) have served as criteria to explain different reactivities. However, comparing the three structures (mal‐, (*S*)‐metmal‐ and (*R*)‐metmal‐Ppant:octanoyl‐KS), we did not find considerable differences in angle or distance that could account for the variations in reactivity observed in the elongation reactions (Figure 3b, Tables S1–S3). Hence, we inspected MD trajectories in criterion (i), the H‐bonds/oxyanion holes of Thr295 and Thr297 as well as His293 and His331 (Table 2). Here, we observed that especially the hydrogen bond probability of the conserved threonines to the thioester oxygen is high for mal‐Ppant but low for (*S*)‐ and (*R*)‐metmal‐Ppant. The hydrogen bond probability is not considerably different between (*S*)‐ and (*R*)‐metmal‐Ppant, suggesting the same reactivity, which is consistent with previous reports of the non‐specific elongation with metmal‐CoA (the stereochemical progression of the putative reaction pathway is discussed in Supporting Information S1) (Kim & Kolattukudy, 1980).

**TABLE 2:** Hydrogen bond probabilities of oxyanion hole residues during five 100 ns trajectories of mal‐ and metmal‐Ppant:KS.

	H‐bond probability (%)			
	Carboxyl group with His		Thioester oxygen with Thr	
	His293	His331	Thr295	Thr297
mal‐Ppant	62.7	50.5	35.4	1.3
(*S*)‐metmal‐Ppant	33.1	33.0	0.2	0.0
(*R*)‐metmal‐Ppant	66.2	23.9	0.0	0.9

To get further insight into the reactivity of the substrates, we selected the center structure with the most appropriate H‐bonds to the histidine and threonine residues, which are referred to in the downstream analysis (Figures S9–S11 and 4b–d). It should be mentioned that the chosen center structures show the acyl group adopting the correct conformation for the condensation reaction, in which the carbonyl group is coordinated in the oxyanion hole of the Cys161 and Phe395 backbone amides. Interestingly, the residue Lys326 sometimes substituted the residue His293 in the carboxyl group coordinating role in the (*S*)‐metmal‐Ppant:KS simulation (Figure 4c).

We questioned why methylmalonyl substrates adopt a slightly different conformation compared to the malonyl conformation. Replacing the pro(*S*) and pro(*R*) H‐atom of the malonyl's C2‐atom without further optimization revealed potential gatekeeper residues. It appears the methyl group of (*S*)‐metmal‐Ppant would clash with the residues of Tyr222 and Met205, while the methyl group of (*R*)‐metmal‐Ppant would clash with the residues of Phe395 and Met205 (Figure S12). We note that Phe395 was identified previously as a potential gatekeeping residue to discriminate against FA cycle intermediates (Rittner et al., 2020). The comparison of mal‐ and metmal‐processing homologous systems does not establish a clear correlation between their specificity and these putative gatekeeper residues (Figure S13).

 .

### 2.4 Mechanistic proposal

**FIGURE 5:**
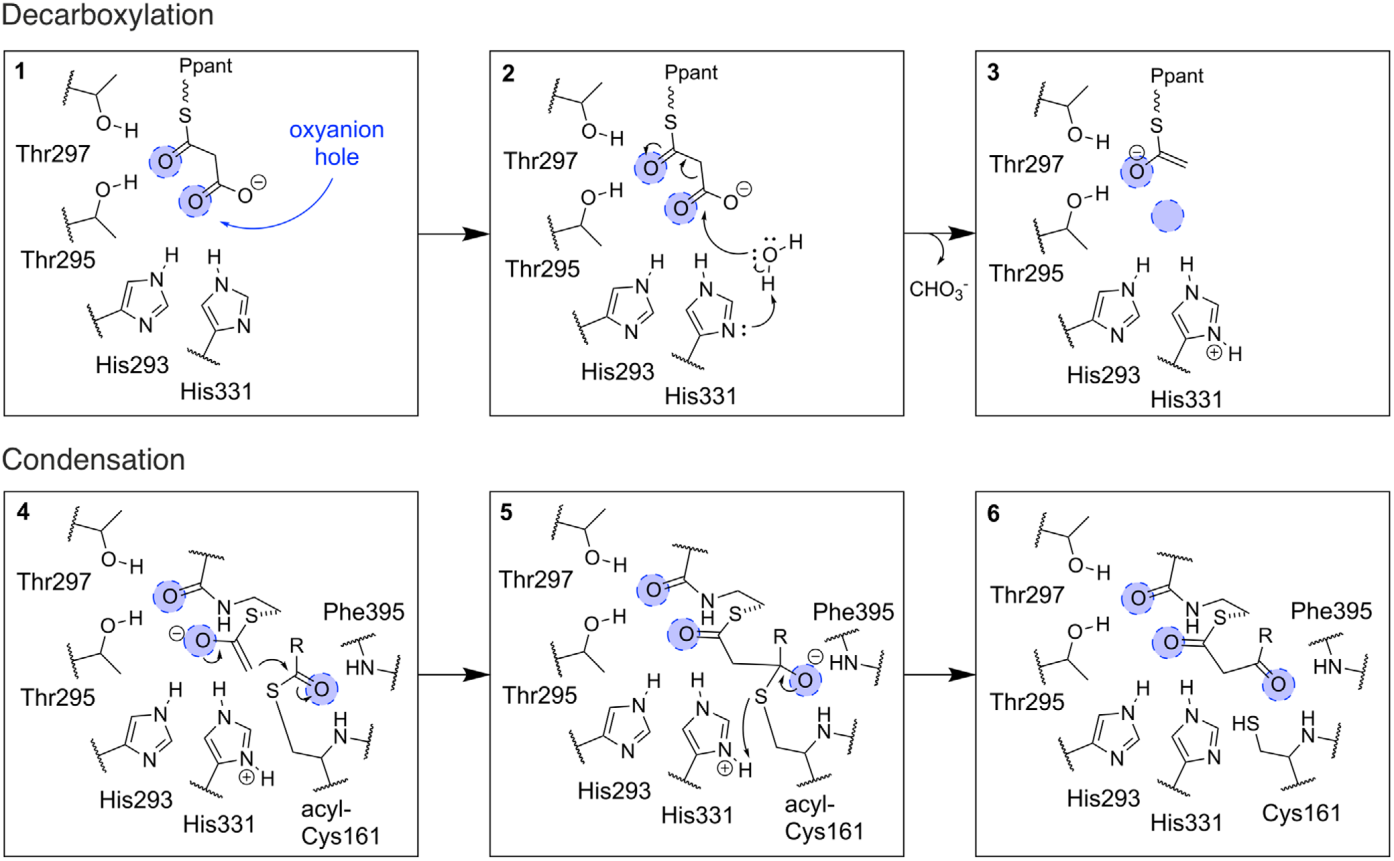
Mechanistic proposal of the decarboxylative condensation reaction. *State 1* represents the binding of the extender substrate. *State 2* shows the decarboxylation reaction. *State 3* bridges the decarboxylation and condensation by allowing a rearrangement of the enolate intermediate. *State 4* shows the condensation mechanism. *State 5* shows the tetrahedral transition state. *State 6* represents the product binding.

Previously discussed decarboxylative condensation mechanisms of KS are not sufficient to explain extender substrate discrimination based on our MD simulations (Paiva et al., 2021). As recent crystallographic studies have provided new insights to extender substrate binding and its decarboxylation reaction in KS(‐like) enzymes (Chisuga et al., 2022), we extended the hitherto mechanistic proposals of the decarboxylative condensation reaction (Figure 5).

The extended model of a six‐step decarboxylative condensation mechanism proceeds to the transacylation of a starter substrate to the active site Cys161.

#### 2.4.1 State 1

Two conserved threonine residues (Thr295 and Thr297) as well as the conserved histidine residues (His293 and His331) form two oxyanion holes, which stabilize (partial) negative charges during the catalysis. Threonine residues stabilize the thioester oxygen, whereas histidine residues coordinate the carboxyl group. This state reflects the crystal structure of the ketosynthase‐like decarboxylase KS^Q^ from *S. graminofaciens* (PDB: 7VEF) (Chisuga et al., 2022).

#### 2.4.2 State 2

During the decarboxylation, water attacks the carboxyl group to form bicarbonate. The fate of the resulting proton is elusive, but it has been suggested that it is not accepted by any of the histidine residues (Lee & Engels, 2013). The thioester oxygen, coordinated in the oxyanion hole of the threonine residues, acts as an electron sink during the decarboxylation. This state reflects that bicarbonate release during the decarboxylation reaction of the rat FAS was determined and a direct formation of bicarbonate during the decarboxylation reaction was proposed (Witkowski et al., 2002). The role of threonine residues agrees with the mutational studies, which revealed their catalytic importance (Chen et al., 2022; Chisuga et al., 2022).

#### 2.4.3 State 3

The resulting negative charge on the thioester oxygen is stabilized by the oxyanion hole of the threonine residues. As the oxyanion hole of the histidine residues is not occupied, the substrate may reach further into the binding pocket of the KS. This state pictures an intermediate stage between the decarboxylation and the condensation. It is important to note that the enolate shift trajectory and timepoint are open to debate, as the movement of the highly reactive intermediate seems unconventional.

#### 2.4.4 State 4

The negative charge of the thioester oxygen is now stabilized by the oxyanion hole of the histidine residues. Alongside the entry of the KS, polar amino acids are thought to guide the prosthetic Ppant group. The oxygen of the amide group in the Ppant is now stabilized by the oxyanion hole of the threonine residues; the reactive enolate intermediate is in proximity to the Cys161‐bound acyl group. The thioester oxygen of the Cys161‐bound acyl group is coordinated in the third oxyanion hole, originating in the coordination of the amide backbones of Cys161 and Phe395. The catalytic role of this oxyanion hole, which also plays a vital role in the transacylation step, is frequently discussed in literature (Heath & Rock, 2002; Paiva et al., 2021; Witkowski et al., 2002). The coordination of the oxyanion holes complies with several crystal structures that capture the KS‐substrate interaction in a close‐to‐catalytic state of the condensation, including, for example, the PKS II ishigamide KS and the FAS II FabB complex (PDB: 6KXF, 5KOF) (Chen et al., 2022; Du et al., 2020; Milligan et al., 2019), as well as the bacterial KSs FabB and FabF with bound cerulenin (Moche et al., 1999; Price et al., 2001).

#### 2.4.5 State 5

The thioester oxygen of the acyl‐Cys161 acts as an electron sink during the nucleophilic attack (condensation) and the resulting negative charge is stabilized by the oxyanion hole. The thioester linkage to Cys161 is cleaved, resulting in the ACP‐bound intermediate. The thiol of Cys161 may or may not be reprotonated at that stage.

#### 2.4.6 State 6

The release‐pathway of the final product (β‐ketoacyl compound) can be guided by the oxyanion holes coordinating the polar oxygens.

## 3 CONCLUSION

The turnover number of BCFA synthesis by mFAS is 150 times lower than the turnover number of StCFA synthesis, demonstrating the preference for mal‐CoA over metmal‐CoA. The low rate is most likely dictated by the KS domain, which shows a slow elongation of FAs with methylmalonyl units. These low elongation rates channel the growing branched acyl chain toward hydrolytic fatty acid release by the thioesterase domain, resulting in reduced product length. The KS rate highly depends on the chain length of the acyl substrate, which indicates that (i) the decarboxylative condensation is influenced by the preceding transacylation and that (ii) the KS might dictate the methylation pattern of the product. The structural analysis of our MD simulations underpins a new mechanistic model in which “oxyanion holes” of threonine and histidine sequentially stabilize intermediate states of decarboxylation and condensation – an arrangement disrupted by the methyl substitution in metmal‐ACP that clashes with potential gatekeeping residues.

We suggest that the engineering of the KS domain can enhance the synthesis of methylated FAs by mFAS, opening new possibilities in using this multienzyme as a biotechnological tool. Additionally, the kinetic data and molecular details of the substrate interaction might contribute to an inhibitor design in the future.

## 4 MATERIALS AND METHODS

### 4.1 Octanoyl‐CoA synthesis

The synthesis of octanoyl‐CoA was performed as described previously (Peter et al., 2015; Valenzano et al., 2010).

In an inert argon atmosphere, 58 μL octanoic acid (0.37 mmol, 6 eq.) was dissolved in 2 mL tetrahydrofuran and cooled to 0°C. Fifty microliters triethylamine (0.37 mmol, 6 eq.) and 28 μL ethylchloroformiate (0.37 mmol, 6 eq.) were added to the solution and stirred for 45 min at 0°C. The reaction mixture was transferred to a 2 mL microcentrifuge tube and centrifuged for 5 min at 20,000 × *g*. The supernatant was transferred to a solution of 50 mg CoA (61 μmol, 1 eq.) dissolved in 2 mL 0.1 M sodium hydrogen carbonate solution. The mixture was stirred for 1 h at room temperature. The reaction mixture was divided, transferred into two 50 mL tubes with 25 mL of cold acetone (−20°C) and centrifuged for 10 min at 10,000 × *g*. After removal of the supernatant, the pellets were transferred to a 1.5 mL microcentrifuge tube and incubated for 15 min at −20°C. The pellet was again centrifuged for 5 min at 20,000 × *g* and dried under reduced pressure. The raw product was obtained as a white solid and stored at −20°C. Since high‐performance liquid chromatography (HPLC) analysis showed a high purity of the synthesized octanoyl‐CoA, no further purification was done (Figure S14).

### 4.2 Preparation of acyl‐ACPs

The functionalized ACPs, acyl‐ACP and metmal‐ACP, served as substrates for the KS‐catalyzed elongation reaction and were used during the MabA assay as described in the section “KS Activity Assay.” The preparation was performed as described previously (Gusenda et al., 2025). In short, unfunctionalized apo‐ACP was catalytically acylated by Sfp and the respective acyl‐CoA. The resulting acyl‐ACP was purified via affinity chromatography using the Strep‐Tactin® XT resin (Figure S15).

### 4.3 Preparation of mFAS

The plasmid containing the full length mFAS construct was transformed into electrically competent *Escherichia coli* BAP1 cells. The transformed cells were plated on an lysogeny broth (LB) agar plate (100 μg mL^−1^ ampicillin, 1% [w/v] glucose) and grown overnight at 37°C. To prepare pre‐cultures, three to six colonies were picked and grown overnight at 37°C and 130 rpm in 20 mL LB medium (100 μg mL^−1^ ampicillin, 1% (w/v) glucose). Pre‐cultures were used to inoculate 1 L terrific broth (TB) medium (100 μg mL^−1^ ampicillin) and grown at 37°C and 130 rpm until they reached an optical density (OD_600_) of 0.6–0.8. The cultures were cooled for 20 min at 4°C, induced with 250 μM isopropyl β‐D‐1‐thiogalactopyranoside (IPTG) and then grown for another 14–16 h at 20°C and 130 rpm. Cells were harvested by centrifugation (4000 × *g* at 4°C for 20 min). The supernatant was discarded and the cell pellets were resuspended in 35 mL lysis buffer (200 mM KCl, 30 mM imidazole, 50 mM potassium phosphate, 10% [v/v] glycerol, 1 mM ethylenediaminetetraacetic acid (EDTA), pH 7.0) and a small amount of DNase I (Sigma Aldrich). The cells were disrupted by the French Pressure Cell Press (Amico) and the resulting suspension was centrifuged (50,000 × *g* at 4°C for 45 min). The supernatant was collected, mixed with 2 mM MgCl_2_ and transferred to Ni‐NTA‐columns. The columns were washed with 5 column volumes (CV) His‐wash buffer (lysis buffer without EDTA), before the bound protein was eluted with 5 CV His‐elution buffer (200 mM KCl, 300 mM imidazole, 50 mM potassium phosphate, 10% [v/v] glycerol, pH 7.0) and collected. The eluate was transferred to Strep‐Tactin® columns and washed with 5 CV Strep‐wash buffer (250 mM potassium phosphate, 1 mM EDTA, 10% [v/v] glycerol, pH 7.0). The bound protein was then eluted with 5 CV Strep‐elution buffer (Strep‐wash buffer with 40 mM biotin) and collected. To monitor the quality of purification, an sodium dodecyl sulfate–polyacrylamide gel electrophoresis (SDS‐PAGE) was performed with samples of every wash and elution step (Figure S16). After concentration to 5–10 mg mL^−1^, the proteins were frozen in liquid nitrogen and stored at −80°C. For further polishing, the proteins were thawed at 37°C for 1 h to enhance dimerization, before being purified via size‐exclusion chromatography with a Superdex 200 GL 10/300 column and Strep‐wash buffer (Figure S17). Fractions with dimeric protein were pooled and concentrated to 15–20 mg mL^−1^. Proteins were aliquoted, frozen in liquid nitrogen and stored at −80°C.

### 4.4 Preparation of KS‐MAT^0^

For the determination of the KS‐catalyzed elongation reaction activity, we expressed the KS‐MAT^S581A^ didomain, with a functional MAT knockout, as the isolated KS domain cannot be produced via recombinant expression in *E. coli*. The protein was expressed in *E. coli* Bl21 gold, purified via affinity chromatography and size‐exclusion chromatography following the same protocol as for the mFAS preparation. All details on protein expression and purification were described previously (Gusenda et al., 2024).

### 4.5 mFAS activity assay

The mFAS activity was measured fluorometrically via NADPH consumption with the *CLARIOstar® Plus Microplate Reader* in 384‐well microplates (Greiner). The assay components were prepared as fourfold stock solutions in activity assay buffer (50 mM potassium phosphate, 10% glycerol [v/v], 5% polyethylene glycol (PEG) 400 [v/v], 1 mM dithiothreitol (DTT), pH 7.0). Stock solutions were pipetted immediately before the measurement to a final assay volume of 20 μL. The final assay solution consisted of 50 μM NADPH, 10–1000 μM acetyl‐CoA, 100 μM metmal‐CoA, and 10 μM mFAS (with 0.03 mg mL^−1^ bovine serum albumin (BSA) for protein stabilization). Addition of metmal‐CoA initiated the enzymatic reaction; the background was monitored through a solution without acetyl‐CoA. The microplate reader was set to measure for 10 min at 25°C with an excitation wavelength of 348–320 nm, emission wavelength of 476–420 nm, gain of 1450, focal height of 12.2, and 70 flashes. Measurements were performed in biological triplicates and analyzed using NADPH calibration at varying concentrations (Figure S18).

### 4.6 KS activity assay

The activity of the KS was determined with the enzyme‐coupled MabA assay, as reported previously (Gusenda et al., 2025). In short, the assay mixture, including 5 μM KS‐MAT^S581A^, 270 μM metmal‐ACP, 0–210 μM acyl‐ACP, 50 μM NADPH, and 5 μM MabA, was prepared in 20 μL of assay buffer (50 mM sodium phosphate, 10% glycerol, pH 7.0) (384‐well plate). The β‐ketoacyl reductase MabA was included in the assay mixture, which reduces the product of the KS reaction (ketoacyl‐ACP) under consumption of NADPH. The fluorescence of NADPH was monitored to follow the elongation reaction of the respective acyl‐ACP with methylmalonyl extender units.

### 4.7 In vitro FA production assay

The assay was performed in 200 μL assay volume with a similar procedure to the mFAS activity assay. Fourfold stock solutions were prepared with activity assay buffer to final concentrations of 1500 μM NADPH, 1000 μM starter substrate (acetyl‐CoA or C8‐CoA), 500 μM metmal‐CoA, and 10 μM mFAS (with 0.03 mg mL^−1^ BSA for protein stabilization). The background was monitored through a solution without CoA‐substrates. After mixing the reaction components, the mixture was incubated overnight at room temperature. As an internal standard, 2 μL heptanoic acid (2 g L^−1^ in methanol/CHCl_3_ 3:1) were added after the incubation phase.

The assay was performed in technical duplicates and biological triplicates.

### 4.8 FA extraction and derivatization

In vitro synthesized FAs were measured via GC–MS to identify the products of the mFAS. To prepare FAs for the measurement, FAs were converted to FA methyl esters (FAMEs), based on a procedure described previously (Bligh & Dyer, 1959).

For this, the assay solution was firstly acidified with 50 μL of 8% HCl in methanol and then mixed with 125 μL MeOH/CHCl_3_ (1:1). The solution was thoroughly shaken to extract FAs into the non‐polar phase. To separate the phases, the solution was centrifuged for 10 min at 3000 × *g*. The non‐polar lower phase was transferred to a 1.5 mL microcentrifuge tube and the solvent was evaporated under low pressure for 15 min. The remains were dissolved in 20 μL toluene and transferred to a Duran glass tube containing 500 μL MeOH and 200 μL 8% HCl in methanol. The solution was sealed, thoroughly shaken, and incubated for 3 h at 100°C to allow methylation. After the incubation, the reaction mixture was cooled on ice for 10 min before the addition of 300 μL H_2_O and 100 μL hexane. The mixture was shaken thoroughly to extract FAMEs into the non‐polar phase. After phase separation, the upper phase was transferred into a GC vial with micro inlet and measured via GC–MS.

### 4.9 GC–MS measurement

FAMEs were measured with the Agilent 7890B gas chromatograph equipped with a HP‐5 ms Ultra Inert capillary column (30 m × 0.25 mm, film thickness: 0.25 μm) and an Agilent 5977B MS detector. The injector temperature was set to 200°C and the detector temperature to 250°C. The temperature program was set as follows: heating to 50°C for 5 min, increasing by 10°C min^−1^ to 120°C, holding for 5 min; increasing by 15°C min^−1^ to 220°C, holding for 10 min.

### 4.10 GC–MS data analysis

The programs OpenChrom, Agilent MassHunter Qualitative Analysis, and NIST MS Search were used to analyze FAME peaks and identify products.

To compensate for product losses during FA extraction and derivatization, the signals were normalized to the signal of the internal standard. The analysis of the data revealed various new peaks above the background. Therefore, a threshold was set to include new signals in the analysis only if they reached at least 5% of the normalized standard peak area. To represent product distribution, the ratios of individual products to total products were calculated.

### 4.11 Docking

The docking of substrate and enzyme was performed on the SwissDock web server (Bugnon et al., 2024) and the AutoDock Vina kernel was selected. The structural data of the dimeric KS was obtained from the PDB database (PDB: 6ROP), and only the KS domain (Positions 1–406) was selected as the target structure for subsequent studies. The coordinates of the box center were located at (94, 94, and 75) and the box size was set to (18) to ensure that the active pocket was completely covered. The sampling exhaustivity was set to the default value of 4.

To select a reasonable conformation from the docking results as the starting point of the MD simulation, the hydrogen bonds between the (methyl)malonyl group and His293, Thr295, Thr297, and His331 were used as the core evaluation indicators. Further, the consistency of the coordinates of the (methyl)malonyl group and the substrate analog in the crystal structure (PDB: 7VEF) was considered.

### 4.12 MD simulation

MD simulations were conducted with GROMACS (version 2022.3) (Abraham et al., 2015) using the Amber99SB‐ildn force field and the TIP3P water model. For the non‐standard residue octanoyl‐Cys161, the force field parameters were optimized by Acpype web tools (Kagami et al., 2023). The amino and carboxyl groups at both ends of independent Cys were saturated with acetyl (ACE) and *N*‐methylformamide (NME) groups, respectively, to fit the peptide bond environment. The *bcc* method was chosen to generate the charge of octanoyl‐Cys161. The multiplicity and net charge of the octanoyl‐Cys161 are 1 and 0, respectively. The atom type of *gaff* is used to specify the atoms in the octanoyl‐Cys161. The force field parameters of substrates were optimized by Acpype web tools. The *bcc* method was chosen to generate the charge of substrates. The atom type of *gaff* is used to specify the atoms in the system. The multiplicity and met charge of substrates are 1 and −3, respectively.

The enzyme was centered in a cubic box with 1 nm between the solute and the box. The system was neutralized with NaCl. Energy minimization was performed applying the steepest descent algorithm until a maximum force of 1000^−1^ kJ mol^−1^ nm^−1^ on any atom was reached. The system was equilibrated by a 1 ns constant particles, volume, temperature (NVT) run at 310 K, followed by a 1 ns constant particles, pressure, temperature (NPT) run at 1 atm and 310 K. Pressure and temperature were controlled using the Velocity‐rescale and Parrinello‐Rahman algorithms. All systems were simulated for 100 ns. Five parallel trajectories were simulated. The five trajectories were processed identically before energy minimization, and each trajectory was subjected to energy minimization and subsequent steps separately.

### 4.13 Trajectory analysis

Trajectories and clustering were performed in Python. MDAnalysis (Michaud‐Agrawal et al., 2011) and scikit‐learn (Pedregosa et al., 2011) packages were used to process the data. The enzyme‐substrate complexes in all trajectories were aligned to the same one‐frame structure as a reference to ensure consistent coordinates during clustering. The clustering analysis selects the coordinates of the malonyl part and the sulfur atom of the substrate. The *K*‐means clustering method was used for clustering. The clustering results were projected to a three‐dimensional principal component analysis. The number of clusters was increased from 3 until all obvious independent point sets in the three‐dimensional projection were divided into different clusters.

## AUTHOR CONTRIBUTIONS

**Christian Gusenda:** Conceptualization; investigation; writing – original draft; visualization; writing – review and editing; formal analysis; supervision. **Kim Ochs:** Investigation; writing – original draft; visualization; writing – review and editing; formal analysis. **Ziheng Cui:** Investigation; formal analysis; writing – original draft; visualization. **Damian L. Ludig:** Formal analysis; supervision; visualization; writing – review and editing. **Martin Grininger:** Conceptualization; supervision; writing – review and editing; funding acquisition.

## CONFLICT OF INTEREST STATEMENT

The authors have no conflict of interest to declare.

## Supporting information


**Data S1.** Supporting Information.

## Data Availability

The data that support the findings of this study are available from the corresponding author upon reasonable request.
