# Supplementary material for: The enzyme kinetics of branched‐chain fatty acid synthesis of metazoan fatty acid synthase
Source: Protein Sci. 2025 Jul 16;34(8):e70229. doi: 10.1002/pro.70229 (PMC12267109; doi:10.1002/pro.70229)
Supplement: Supplementary file 1 — Data S1. Supporting Information. [file PRO-34-e70229-s001.zip › si_gusenda&ochs.pdf]

Supporting Information

The enzyme kinetics of branched-chain fatty acid synthesis of metazoan fatty acid synthase

Christian Gusenda[1,*], Kim Ochs[1,*], Ziheng Cui[1], Damian L. Ludig[1], Martin Grininger[1]

[1] Institute of Organic Chemistry and Chemical Biology
Buchmann Institute of Molecular Life Sciences, Goethe University Frankfurt
Max-von-Laue-Str. 15, 60438 Frankfurt am Main, Germany

* Authors contributed equally to this work.

Correspondence

Christian Gusenda, Institute of Organic Chemistry and Chemical Biology
Buchmann Institute of Molecular Life Sciences, Goethe University Frankfurt
Max-von-Laue-Str. 15, 60438 Frankfurt am Main, Germany
Email: gusenda@biochem.uni-frankfurt.de

Martin Grininger, Institute of Organic Chemistry and Chemical Biology
Buchmann Institute of Molecular Life Sciences, Goethe University Frankfurt
Max-von-Laue-Str. 15, 60438 Frankfurt am Main, Germany
Email: grininger@chemie.uni-frankfurt.de

Equations describing enzyme kinetics:

(1) Michealis-Menten $v = \frac{v_{max} \cdot [S]}{K_m + [S]}$

(2) Hill equation (Cooperativity) $v = \frac{v_{max} \cdot [S]^h}{K'^h + [S]^h}$

(3) Substrate Inhibition $v = \frac{v_{max} \cdot [S]}{K_m + [S] + \frac{[S]^2}{K_i}}$

(4) Cooperativity + Substrate Inhibition $v = \frac{v_{max} \cdot [S]^h}{K'^h + [S]^h + \frac{[S]^{2h}}{K_i^h}}$

where: v is the reaction velocity, v_{max} is the maximal reaction velocity, *[S]* is the substrate concentration, K_m is the Michaelis-Menten constant, h is the Hill coefficient, K' (also commonly known as $K_{0.5}$) is the half-concentration constant and K_i is the inhibition constant.

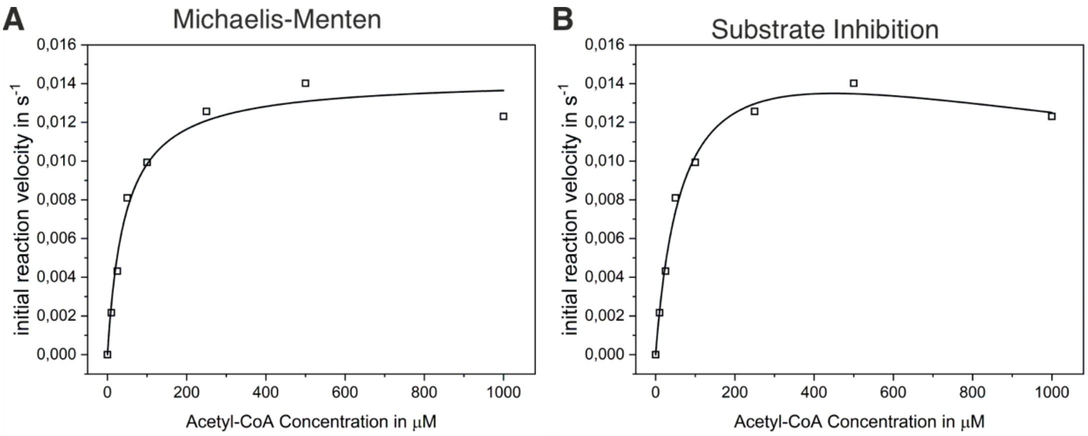

Figure S1: The titration curve of acetyl-CoA during the mFAS activity assay fitted with different kinetic models. **A** Fit under assumption of regular steady-state kinetics with Michalis-Menten equation 1 (MM). **B** Fit under assumption of substrate inhibition with equation 3. The F-Test on the basis of the residual sum of squares resulted in a *p*-value of $p = 0.04$, indicating a reasonably better description of the kinetic data under assumption of substrate inhibition (eq. 3).

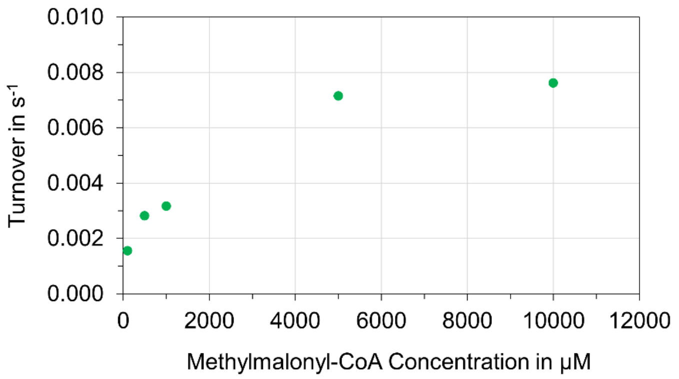

Figure S2: Self-Priming of (*R,S*)-metmal-CoA. The mFAS activity assay was performed as described in the method section, with the following components: 50 µM NADPH, 10 µM mFAS (with 0.03 mg mL^{-1} BSA) and varying concentrations of (*R,S*)-metmal-CoA. The assay was performed without any starter substrate to measure the self-priming effect of metmal-CoA.

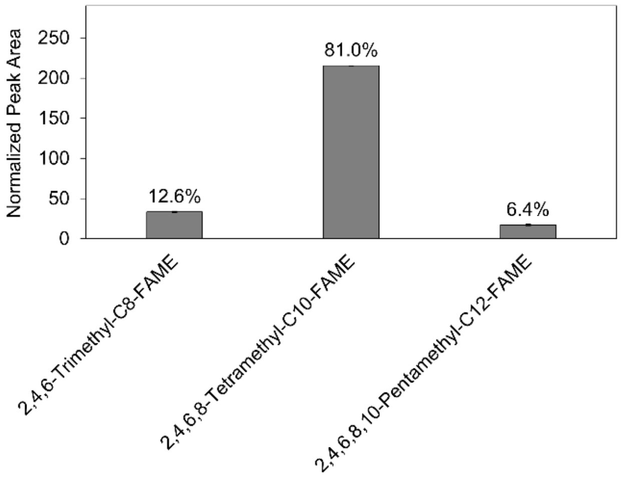

Figure S3: Product distribution of mFAS with acetyl-CoA and (*R,S*)-metmal-CoA. The bars represent peak areas normalized to the peak area of the internal standard, whereas numbers above the bars indicate ratios of the individual products to total products. The products were enzyme-catalytically synthesized overnight and transformed into fatty acid methyl esters before monitoring in GC-MS, as described in the method section. Here, the threshold was changed to include signals above the background, if they reached at least 1.5% of the normalized standard peak area. The bars represent means of two technical replicates with error bars representing the standard deviation.

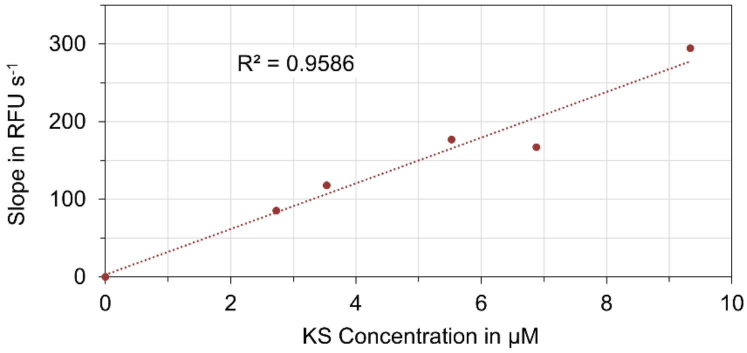

Figure S4: KS titration. The MabA assay was performed as described in section “KS Activity Assay” with the following components: 150 µM C10-ACP, 250 µM metmal-ACP and varying concentrations of KS. As the activity is proportional to the enzyme concentration over the range of 0-9.3 µM KS, the readout is not limited by the coupled enzyme reaction at 5 µM KS.

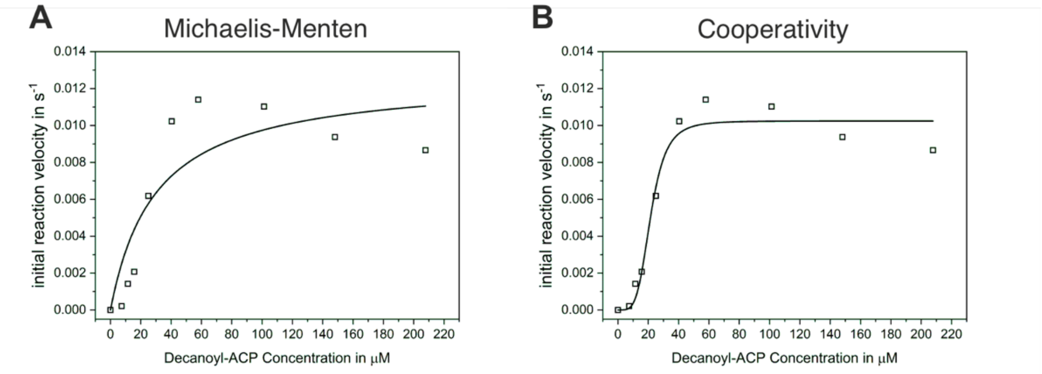

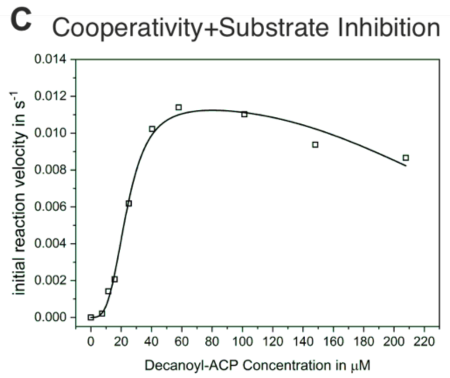

D

Model	MM	Coop.
Coop.	0.0006 (***)	
Coop.+Inhib.	0.00005 (***)	0.004 (**)

Figure S5: Titration curve of decanoyl-ACP during the MabA assay fitted with different kinetic models. **A** Fit under assumption of regular steady-state kinetics with MM equation. **B** Fit under assumption of cooperative kinetics with Hill equation. **C** Fit under assumption of cooperative kinetics and substrate inhibition with equation 4. **D** Comparison of different kinetic models by F-Test derived *p*-values. The first column shows the more complex model (more parameters) compared with the more basic model (less parameters) in the first row. Note: A fit with the equation for substrate inhibition (eq. 3) did not converge. OriginPro 2023 was used for data fitting. Data points represent the means of three biological replicates. Asterisks mark the significance: ** $p < 0.01$; *** $p < 0.001$.

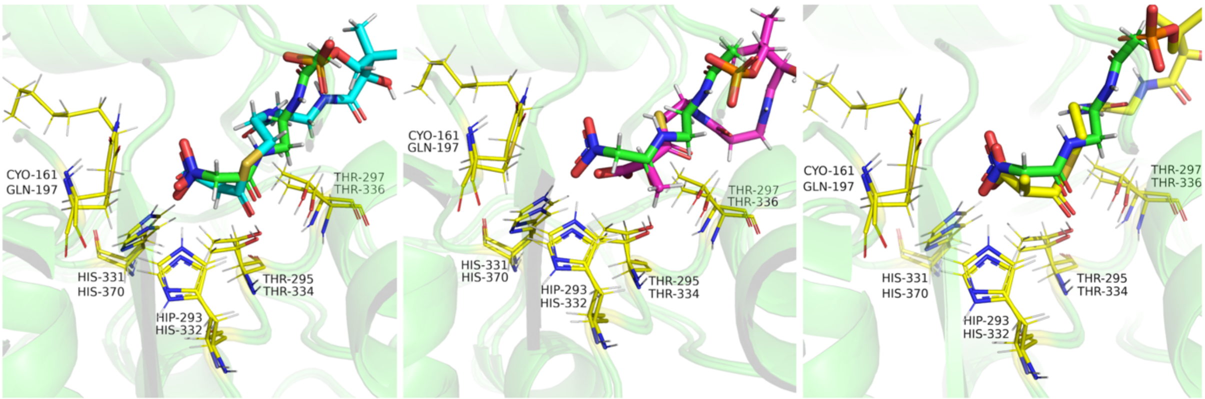

Malonyl No. 16
Cyan

R-methymalonyl
No. 10
Purple

S-methymalonyl
No. 19
Yellow

Figure S6: Docking results. The 20 best docking results of substrates with murine octanoyl-KS (PDB:6ROP) by SwissDock were judged in comparison with the crystal structure of nitroacetyl pantetheinamide:KS_Q (PDB: 7VEF). The reference substrate conformation is shown in green, whereas the docked substrates are shown in cyan (mal), magenta ((*R*)-metmal) and yellow ((*S*)-metmal), respectively.

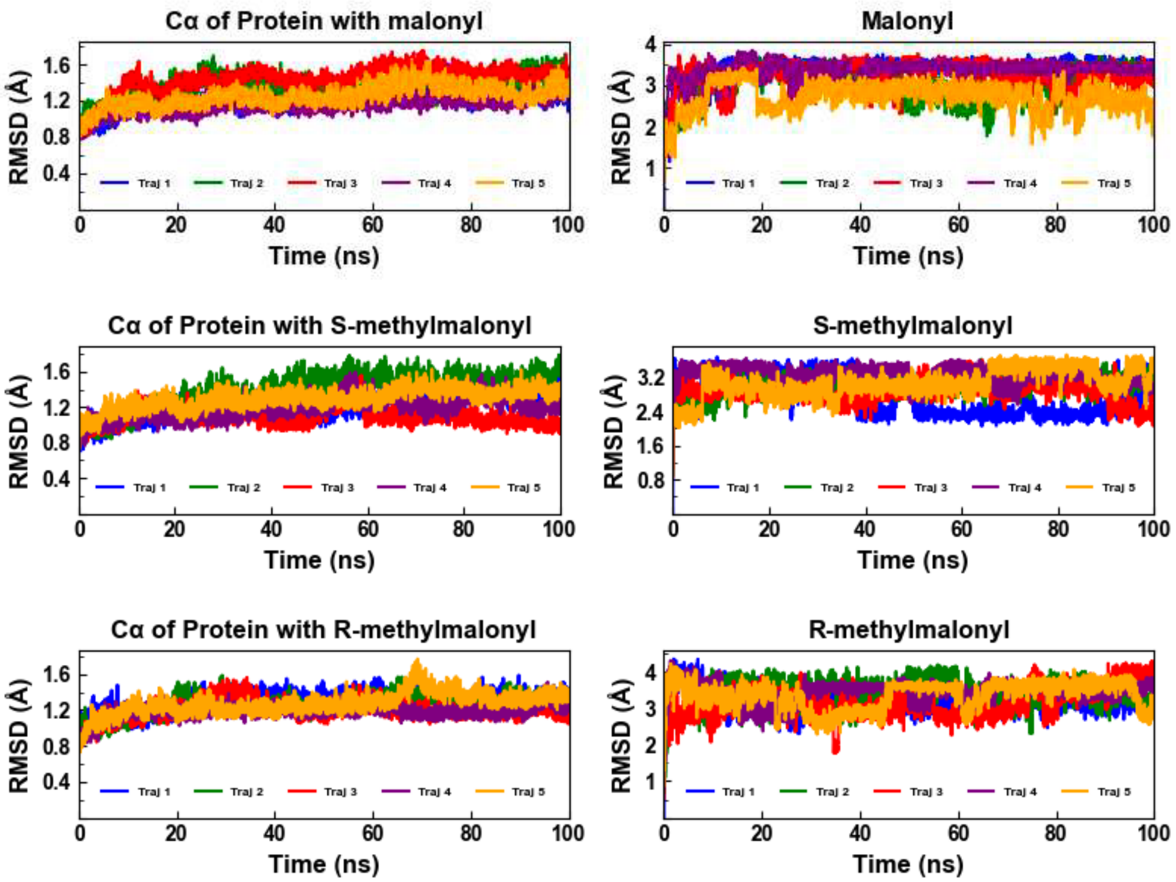

Figure S7: The RMSD values of the Cα-atoms of the protein backbone (left) and all atoms of the substrate (right) during 100 ns simulation. The values for five independent trajectories are shown (Traj 1-5). For downstream analysis, frames between 40-100 ns were considered.

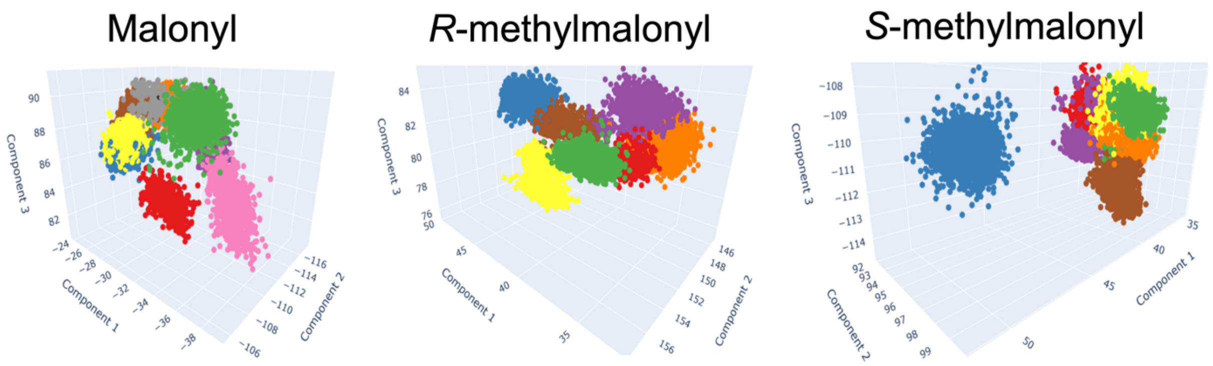

Figure S8: Clustering using the K-means clustering method. The clustering results are projected under three principal components. The number of clusters is increased until all clusters of the three-dimensional projection space are visualized independently. As a result, nine clusters of the malonyl MD simulation and seven clusters of the (*S*)- and (*R*)-methylmalonyl MD simulations were identified respectively.

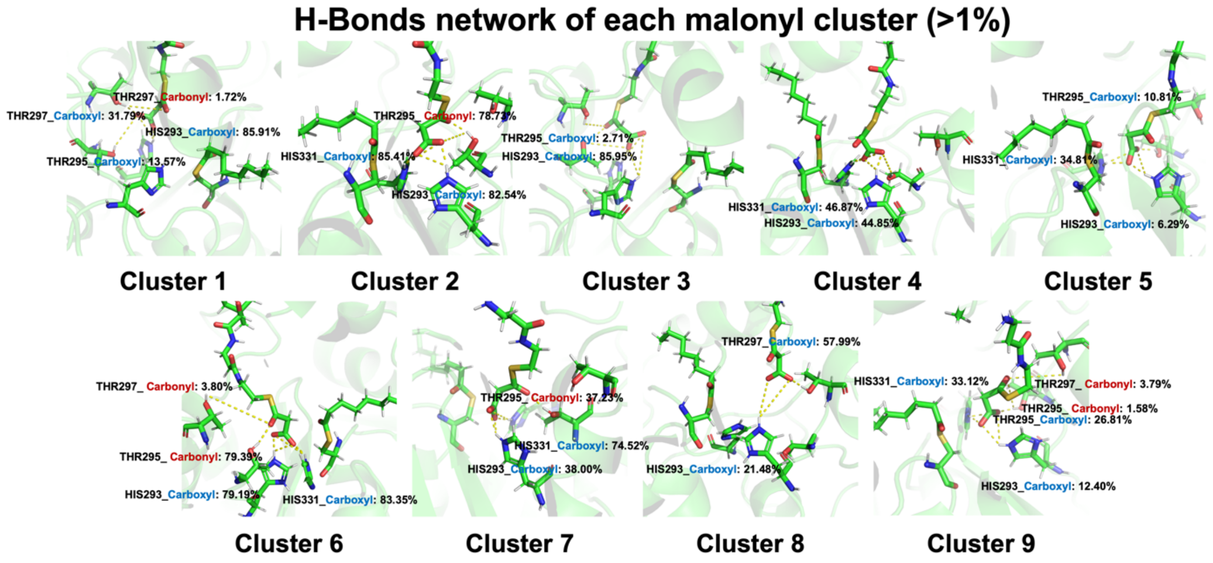

Figure S9: Visual depiction of the center structure in each cluster of the malonyl MD simulation. The center structure of cluster 6 shows the H-bonds to the conserved histidine and threonine residues and is considered the closest to a catalytic state.

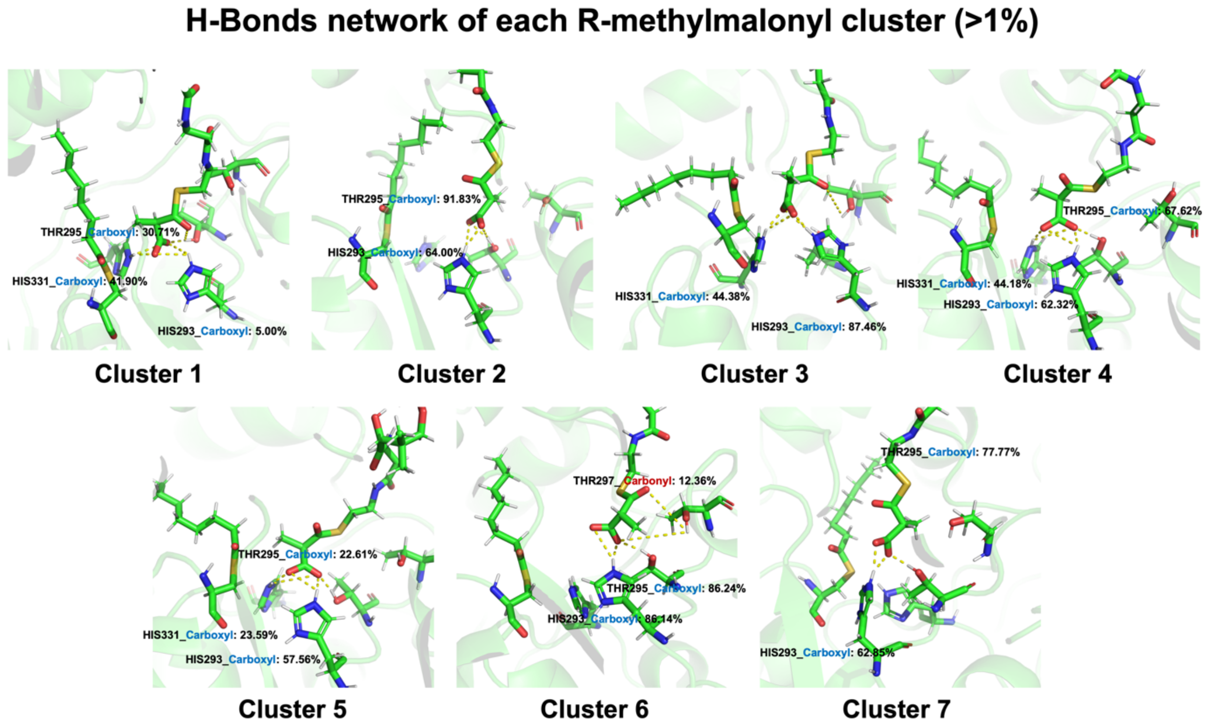

Figure S10: Visual depiction of the center structure in each cluster of the (*S*)-methylmalonyl MD simulation. The center structure of cluster 7 shows the H-bonds to the conserved histidine and threonine residues and is considered the closest to a catalytic state.

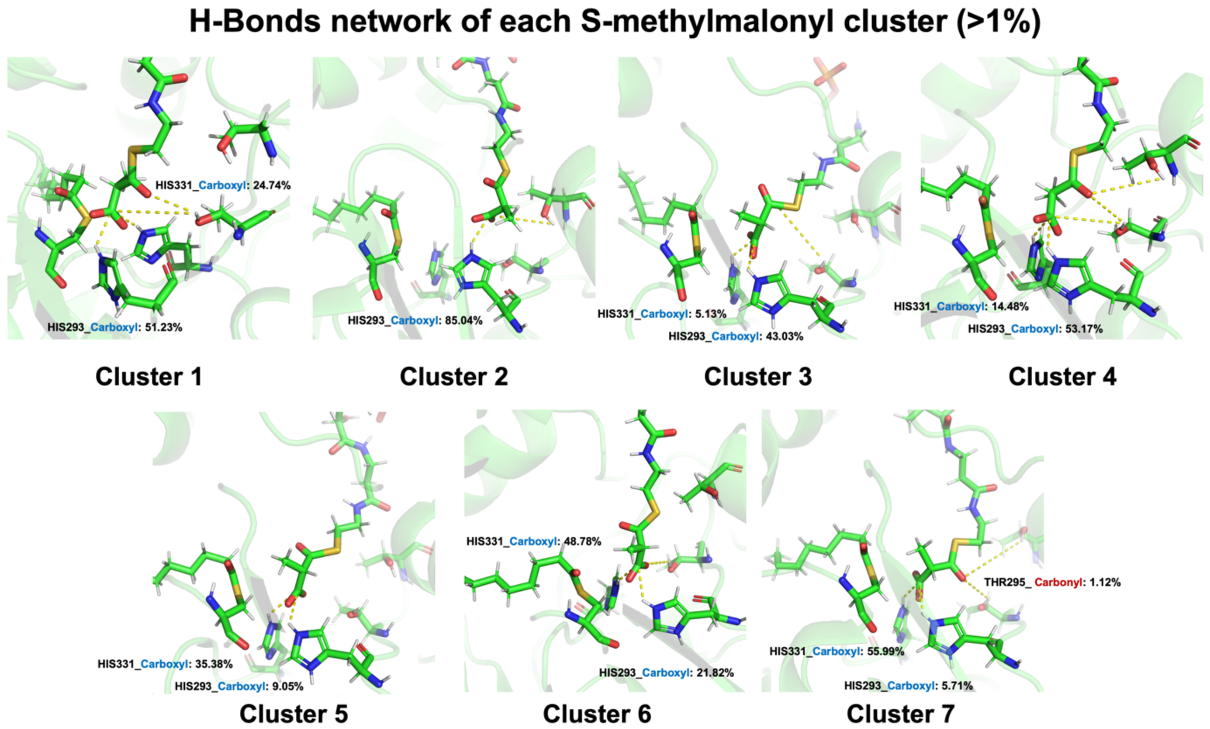

Figure S11: Visual depiction of the center structure in each cluster of the (*R*)-methylmalonyl MD simulation. The center structure of cluster 3 shows the H-bonds to the conserved histidine and one threonine residues and is considered the closest to a catalytic state.

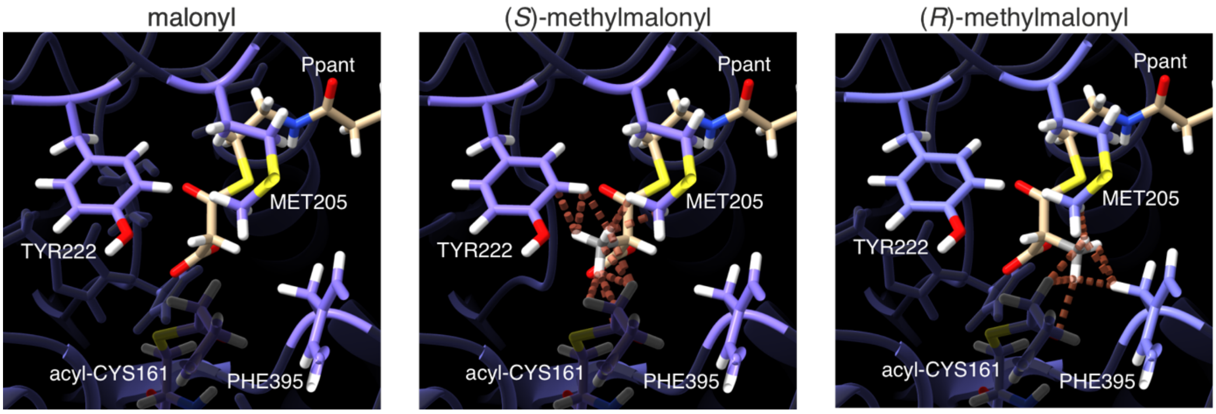

Figure S12: Clashes of methyl groups. A methyl group was modeled into the center structure of mal-Ppant:octanoyl-KS replacing the pro(*S*)- and pro(*R*)-hydrogen atom, respectively, without any optimization. The substrate mal-Ppant is shown in beige; the methylgroup is shown in grey. Clashes were identified below atom-atom distances of 2.4 Å and shown with orange dashed lines.

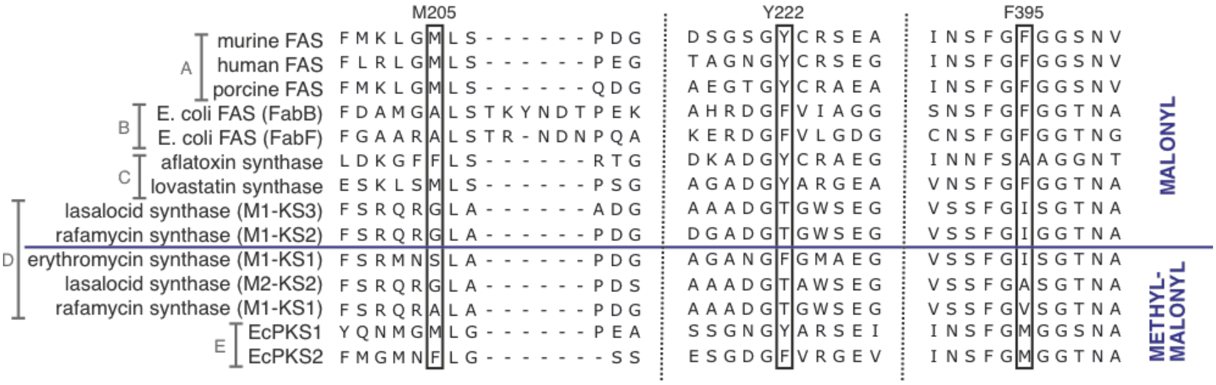

Figure S13: Sequence alignment of KS domains from different biosynthetic systems. The putative gatekeeping residues M205, Y222, F395 (murine numbering) are highlighted. Malonyl-processing domains include KS from A: mammalian FAS, B: bacterial FAS, C: iterative PKS, D: modular PKS. Methylmalonyl-processing domains include KS from D: modular PKS and E: animal FAS-like PKS.

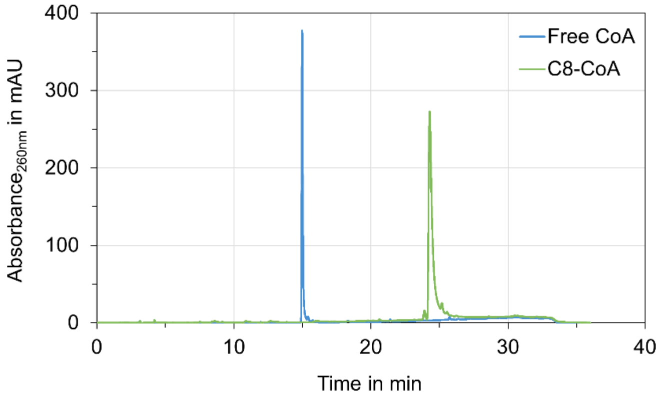

Figure S14: HPLC analysis of octanoyl-CoA and free CoA. The HPLC measurement was performed with 200 mM ammonium acetate buffer (pH 6.0) and a gradient starting at 5% methanol and increasing until 90% methanol. As the absorbance at 260 nm showed no free CoA in the synthesized octanoyl-CoA, no further purification steps were carried out after the synthesis.

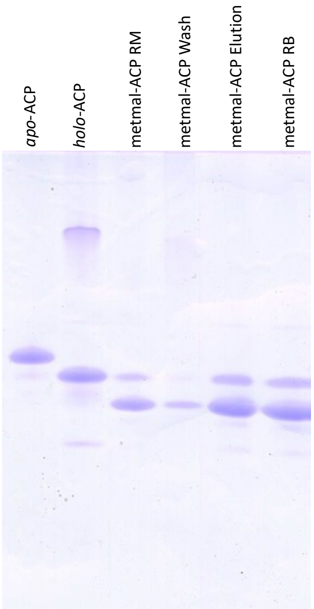

Figure S15: Urea gel of metmal-ACP. The apo-ACP expression, acylation, purification and analysis was described previously in detail.[72] *apo*-ACP and *holo*-ACP are shown as reference. RM: reaction mix of the acylation reaction, wash: wash fraction of Strep-Tactin® based affinity chromatography, elution: elution-fraction of Strep-Tactin® based affinity chromatography, RB: rebuffered sample after buffer exchange through Amicon® centrifugal filters. The final yield of metmal-ACP was estimated with ImageJ to about 80%.[1]

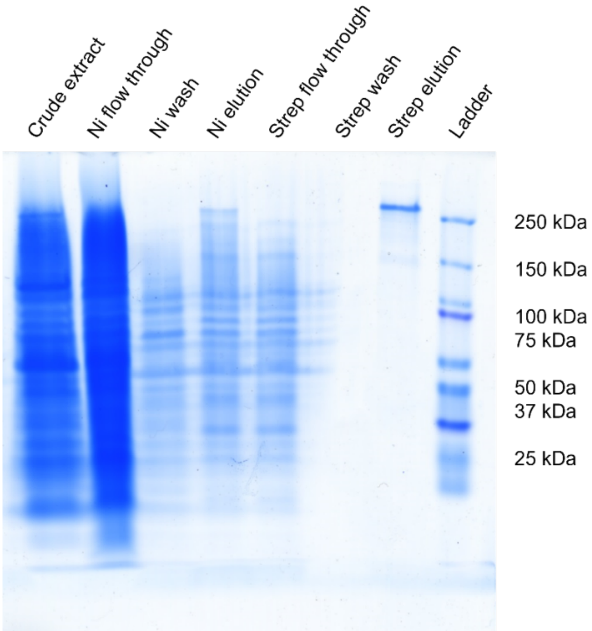

Figure S16: Representative SDS-PAGE analysis of the full length mFAS produced in *E. coli*. Apparent molecular weight of the target protein in the Strep elution fraction is consistent with the calculated molecular weight of 270 kDa.

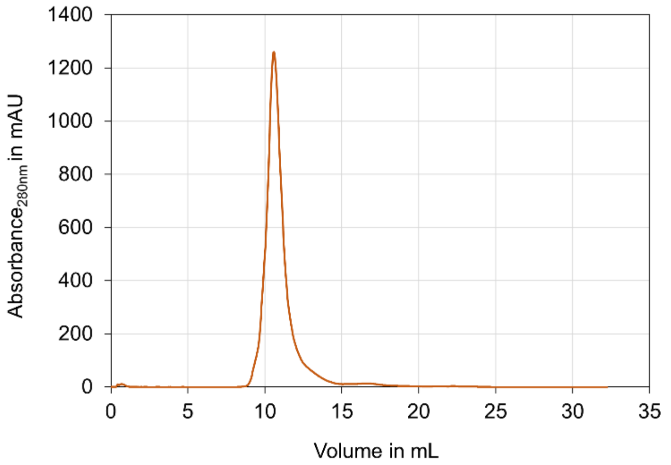

Figure S17: Representative size-exclusion chromatogram of the full length mFAS with the Superdex 200 GL 10/300 column. The chromatogram shows a prominent peak at 10.5 mL elution volume, representing the preferential dimeric state of the mFAS. Fractions of dimeric proteins were collected and used for further experiments.

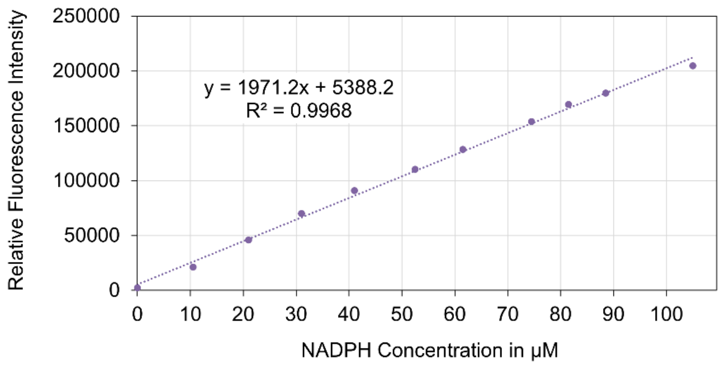

Figure S18: NADPH calibration. The relative fluorescence intensity of NADPH at different concentrations was measured using the settings described in the section "mFAS Activity Assay". The reaction mixture contained NADPH at varying concentrations and 0.03 mg mL^{-1} BSA. The data points represent means of technical triplicates. The data was fitted via linear regression; the linear equation as well as the coefficient of determination are displayed.

Table S1: Data from mal-Ppant:octanoyl-KS MD simulation. Frame ratio of each cluster, H-bond probability of residues highlighted in Figure 4, dihedral angle relevant for decarboxylation readiness and distance between mal-C2 and octanoyl-C3 relevant for the proceeding condensation reaction. Values of representative cluster 6 are shown in green.

Cluster No.	Frame ratio	H-Bonds probability (%): Carboxyl group with two HIS		H-Bonds probability (%): Thioester group with two THR		Dihedral (Degree)	Err	Distance (Å)	Err
		HIS293	HIS331	THR295	THR297				
1	4%	85,91	-	-	1,72	31,63	7,96	7,14	0,09
2	13%	82,54	85,41	78,73	-	67,33	0,63	4,19	0,02
3	19%	85,95	0,35	0,04	0,88	56,42	9,59	7,06	0,06
4	12%	44,85	46,87	-	-	51,32	1,68	4,06	0,03
5	8%	6,29	34,81	-	-	59,42	0,52	3,95	0,01
**6**	**26%**	**79,19**	**83,35**	**79,39**	**3,80**	**50,58**	**2,51**	**3,95**	**0,03**
7	11%	38,00	74,52	37,23	0,06	57,88	0,97	3,80	0,01
8	5%	21,48	-	-	-	21,92	5,09	6,62	0,34
9	2%	12,30	33,12	1,58	3,79	66,73	6,58	4,68	0,17

Table S2: Data from (*S*)-metmal-Ppant:octanoyl-KS MD simulation. Frame ratio of each cluster, H-bond probability of residues highlighted in Figure 4, dihedral angle relevant for decarboxylation readiness and distance between mal-C2 and octanoyl-C3 relevant for the proceeding condensation reaction. Values of representative cluster 3 are shown in green.

Cluster No.	Frame ratio	H-Bonds probability (%) Carboxyl group with two HIS		Thioester group with two THR		Dihedral (Degree)	Err	Distance (Å)	Err
		HIS293	HIS331	THR295	THR297				
1	3%	5,00	41,90	0,24	-	22,19	1,46	5,44	0,04
2	20%	64,00	-	-	-	37,29	0,69	7,78	0,05
**3**	**18%**	**87,46**	**44,38**	**-**	**0,18**	**49,89**	**0,91**	**4,01**	**0,01**
4	21%	62,32	44,18	-	-	72,88	2,77	5,04	0,04
5	22%	57,56	23,59	-	-	76,11	0,64	5,19	0,01
6	7%	86,14	-	-	12,36	105,60	22,30	7,69	0,02
7	9%	62,85	-	-	-	31,95	0,60	8,35	0,25

Table S3: Data from (*R*)-metmal-Ppant:octanoyl-KS MD simulation. Frame ratio of each cluster, H-bond probability of residues highlighted in Figure 4, dihedral angle relevant for decarboxylation readiness and distance between mal-C2 and octanoyl-C3 relevant for the proceeding condensation reaction. Values of representative cluster 7 are shown in green.

Cluster No.	Frame ratio	H-Bonds probability (%) Carboxyl group with two HIS		Thioester group with two THR		Dihedral (Degree)	Err	Distance (Å)	Err
		HIS293	HIS331	THR295	THR297				
1	11%	51,23	24,74	0,23	-	80,41	0,66	4,29	0,05
2	20%	85,04	-	-	-	80,19	0,91	8,29	0,02
3	16%	5,13	43,03	0,13	-	89,77	8,18	4,63	0,03
4	6%	53,17	14,48	0,34	0,11	41,43	2,56	4,08	0,02
5	11%	9,05	35,38	-	-	78,26	2,69	4,68	0,02
6	20%	21,82	48,78	-	-	62,72	0,94	4,75	0,02
**7**	**15%**	**5,71**	**55,99**	**1,12**	**0,17**	**50,71**	**4,54**	**4,75**	**0,02**

Discussion of stereochemical aspects

We questioned whether we could predict the stereochemistry of the elongation product based on our MD simulations. As the enolate intermediate of the condensation reaction does not bear any stereochemical information, the configuration of the product is determined by the geometry of the binding site and the direction of the nucleophilic attack. If we assume no/minimal rearrangement of the (*S*)-metmal-residue and the resulting enolate intermediate, the subsequent nucleophilic attack during the condensation reaction would likely proceed under retention from the re-face of the enolate resulting in a (*S*)-methyl branching of the BCFA. This interpretation contradicts previous studies, which found an inversion during the condensation of malonyl units in mFAS,[2] as well as during the condensation of (*S*)-metmal-units in PKS.[3] This contradiction can be resolved by assuming a rearrangement of the enolate intermediate after decarboxylation, as discussed in the main text (Figure 4A, State 3-4). The same speculation cannot be made for the (*R*)-metmal substrate, as it is not possible to determine the most probable face of nucleophilic attack. We acknowledge that these considerations are hypothetical, but MD simulations of the enolate intermediate in the binding site would allow more precise predictions.

Protein Sequences

Tag and linker sequences are highlighted in cyan.

Amino acid sequence of mFAS

MSAWSHPQFEKGGGSGGGSGGSAWSHPQFEKGAGSEEVVIAGMSGKLPESENLQEFWANLIGGV
DMVTDDDRRWKAGLYGLPKRSGKLKDLSKFDASFFGVHPKQAHTMDPQLRLLLEVSYEAIVDGGINP
ASLRGTNTGVWVGVSGSEASEALSRDPETLLGYSMVGCQRAMMANRLSFFFDFKGPSIALDTACSS
SLLALQNAYQAIRSGECPAALVGGINLLLKPNTSVQFMKLGMLSPDGTCRSFDDSGSGYCRSEAVVA
VLLTKKSLARRVYATILNAGTNTDGSKEQGVTFPSGEVQEQLICSLYQPAGLAPESLEYIEAHGTGTKV
GDPQELNGITRSLCAFRQAPLLIGSTKSNMGHPEPASGLAALTKVLLSLEHGVWAPNLHFHNPNPEIP
ALLDGRLQVVDRPLPVRGGNVGINSFGFGGSNVHVILQPNTRQAPAPTAHAALPHLLHASGRTLEAV
QDLLEQGRQHSQDLAFVSMLNDIAATPTAAMPFRGYTVLGVEGRVQEVQQVSTNKRPLWFICSGMG
TQWRGMGLSLMRLDSFRESILRSDEAVKPLGVKVSDLLLSTDERTFDDIVHAFVSLTAIQIALIDLLTSV
GLKPDGIIGHSLGEVACGYADGCLSQREAVLAAYWRGQCIKDAHLPPGSMAAVGLSWEECKQRCPA
GVVPACHNSEDTVTISGPQAAVNEFVEQLKQEGVFAKEVRTGGLAFHSYFMEGIAPTLLQALKKVIRE
PRPRSARWLSTSIPEAQWQSSLARTSSAEYNVNNLVSPVLFQEALWHIPEHAVVLEIAPHALLQAVLK
RGVKSSCTIIPLMKRDHKDNLEFFLTNLGKVHLTGINVNPNALFPPVEFPAPRGTPLISPHIKWDHSQT
WDVPVAEDFPNGSSSSSATVYSIDASPESPDHYLVDHCIDGRVIFPGTGYLCLVWKTLARSLGLSLEE
TPVVFENVSFHQATILPKTGTVALEVRLLEASHAFEVSDTGNLIVSGKVYLWEDPNSKLFDHPEVPTPP
ESASVSRLTQGEVYKELRLRGYDYGPQFQGICEATLEGEQGKLLWKDNWVTFMDTMLQVSILGSSQ
QSLQLPTRVTAIYIDPATHRQKVYRLKEDTQVADVTTSRCLGITVSGGIHISRLQTTATSRRQQEQLVP
TLEKFVFTPHMEAECLSESTALQKELQLCKGLARALQTKATQQGLKAAMLGQEDPPQHGLPRLLAAA
CQLQLNGNLQLELGEALAQERLLLPEDPLISGLLNSQALKACVDTALENLSTLKMKVAEVLAGEGHLY
SRIPALLNTQPMLQLEYTATDRHPQALKDVQTKLQQHDVAQGQWNPSDPAPSSLGALDLLVCNCALA
TLGDPALALDNMVAALKEGGFLLVHTVLKGHALGETLACLPSEVQPAPSLLSQEEWESLFSRKALHLV
GLKRSFYGTALFLCRRAIPQEKPIFLSVEDTSFQWVDSLKSTLATSSSQPVWLTAMDCPTSGVVGLVN
CLRKEPGGHRIRCILLSNLSNTSHAPKLDPGSPELQQVLKHDLVMNVYRDGAWGAFRHFQLEQDKPK
EQTAHAFVNVLTRGDLASIRWVSSPLKHTQPSSSGAQLCTVYYASLNFRDIMLATGKLSPDAIPGKWA
SRDCMLGMEFSGRDRCGRRVMGLVPAEGLATSVLLSSDFLWDVPSSWTLEEAASVPVVYTTAYYSL
VVRGRIQRGETVLIHSGSGGVGQAAISIALSLGCRVFTTVGSAEKRAYLQARFPQLDDTSFANSRDTS
FEQHVLLHTGGKGVDLVLNSLAEEKLQASVRCLAQHGRFLEIGKFDLSNNHPLGMAIFLKNVTFHGILL
DALFEEANDSWREVAALLKAGIRDGVVKPLKCTVFPKAQVEDAFRYMAQGKHIGKVLVQVREEEPEA
VLPGAQPTLISAISKTFCPAHKSYIITGGLGGFGLELARWLVLRGAQRLVLTSRSGIRTGYQAKHIREW
RRQGIQVLVSTSNVSSLEGARALIAEATKLGPVGGVFNLAMVLRDAMLENQTPELFQDVNKPKYNGTL
NLDRATREACPELDYFVAFSSVSCGRGNAGQTNYGFANSTMERICEQRRHDGLPGLAVQWGAIGDV
GIVLEAMGTNDTVIGGTLPQRISSCMEVLDLFLNQPHAVLSSFVLAEKKAVAHGDGDTQRDLVKAVAHI
LGIRDLAGINLDSTLADLGLDSLMGVEVRQILEREHDLVLPMREVRQLTLRKLQEMSSKTDSATDTTAP
KSRSDTSLKQNQLNLSTLLVNPEGPTLTQLNSVQSSERPLFLVHPIEGSTTVFHSLAAKLSVPTYGLQC
TQAAPLDSIPNLAAYYIDCIKQVQPEGPYRIAGYSFGACVAFEMCSQLQAQQGPAPTHNNLFLFDGSH
TYVLAYTQSYRAKMTPGCEAEAEAEALCFFIKQFLDVEHSKVLEALLPLKSLEDRVAASVDLITKSHHS
LDRRELSFAAVSFYHKLRAADQYKPKAKYHGNVTLLRAKTGGTYGEDLGADYNLSQVCDGKVSVHIIE
GDHRTLLEGSGLESIINIIHSSLAEPRVSVREGLEHHHHHHHH

The plasmid is based on the pET22b vector and was prepared by Alexander Rittner.

Amino acid sequence of KS-MAT^{S581A}

MSAWSHPQFEKGGGSGGGSGGSAWSHPQFEKGAGSEEVVIAGMSGKLPESENLQEFWANLIGGV
DMVTDDDRRWKAGLYGLPKRSGKLKDLSKFDASFFGVHPKQAHTMDPQLRLLLEVSYEAIVDGGINP
ASLRGTNTGVWVGVSGSEASEALSRDPETLLGYSMVGCQRAMMANRLSFFFDFKGPSIALDTACSS
SLLALQNAYQAIRSGECPAALVGGINLLLKPNTSVQFMKLGMLSPDGTCRSFDDSGSGYCRSEAVVA
VLLTKKSLARRVYATILNAGTNTDGSKEQGVTFPSGEVQEQLICSLYQPAGLAPESLEYIEAHGTGTKV
GDPQELNGITRSLCAFRQAPLLIGSTKSNMGHPEPASGLAALTKVLLSLEHGVWAPNLHFHNPNPEIP
ALLDGRLQVVDRPLPVRGGNVGINSFGFGGSNVHVILQPNTRQAPAPTAHAALPHLLHASGRTLEAV
QDLLEQGRQHSQDLAFVSMLNDIAATPTAAMPFRGYTVLGVEGRVQEVQQVSTNKRPLWFICSGMG
TQWRGMGLSLMRLDSFRESILRSDEAVKPLGVKVSDLLLSTDERTFDDIVHAFVSLTAIQIALIDLLTSV
GLKPDGIIGHALGEVACGYADGCLSQREAVLAAYWRGQCIKDAHLPPGSMAAVGLSWEECKQRCPA
GVVPACHNSEDTVTISGPQAAVNEFVEQLKQEGVFAKEVRTGGLAFHSYFMEGIAPTLLQALKKVIRE
PRPRSARWLSTSIPEAQWQSSLARTSSAEYNVNNLVSPVLFQEALWHIPEHAVVLEIAPHALLQAVLK
RGVKSSCTIIPLMKRDHKDNLEFFLTNLGKVHLTGINVNPNALFPPVEFPAPRGTPLISPHIKWDHSQT
WDVPVAEDFPNGSGSPSAHHHHHHHH

The plasmid is based on the pET22b vector and was prepared by Alexander Rittner.

References

1. Schneider CA, Rasband WS, Eliceiri KW (2012) NIH Image to ImageJ: 25 years of image analysis. Nat Methods 9:671–675.

2. Anderson VE, Hammes GG (1984) Stereochemistry of the reactions catalyzed by chicken liver fatty acid synthase. Biochemistry 23:2088–2094.

3. Weissman KJ, Timoney M, Bycroft M, Grice P, Hanefeld U, Staunton J, Leadlay PF (1997) The Molecular Basis of Celmer's Rules: The Stereochemistry of the Condensation Step in Chain Extension on the Erythromycin Polyketide Synthase. Biochemistry 36:13849–13855.
